# Evidence for a Common Origin of Homomorphic and Heteromorphic Sex Chromosomes in Distinct *Spinacia* Species

**DOI:** 10.1534/g3.115.018671

**Published:** 2015-06-05

**Authors:** Satoshi Fujito, Satoshi Takahata, Reimi Suzuki, Yoichiro Hoshino, Nobuko Ohmido, Yasuyuki Onodera

**Affiliations:** * Research Faculty of Agriculture, Hokkaido University, N-9, W-9, Sapporo 060-8589, Japan; † Field Science Center for Northern Biosphere, Hokkaido University, N-11, W-10, Sapporo 060-0811, Japan; ‡ Graduate School of Human Development and Environment, Kobe University, Kobe 657-8501, Japan

**Keywords:** *Spinacia*, spinach, dioecy, sex chromosome, nuclear DNA amount, genetics of sex

## Abstract

The dioecious genus *Spinacia* is thought to include two wild relatives (*S*. *turkestanica* Ilj. and *S*. *tetrandra* Stev.) of cultivated spinach (*S*. *oleracea* L.). In this study, nuclear and chloroplast sequences from 21 accessions of *Spinacia* germplasm and six spinach cultivars or lines were subjected to phylogenetic analysis to define the relationships among the three species. Maximum-likelihood sequence analysis suggested that the *Spinacia* plant samples could be classified into two monophyletic groups (Group 1 and Group 2): Group 1 consisted of all accessions, cultivars, and lines of *S*. *oleracea* L. and *S*. *turkestanica* Ilj. and two of five *S*. *tetrandra* Stev. accessions, whereas Group 2 was composed of the three remaining *S*. *tetrandra* Stev. accessions. By using flow cytometry, we detected a distinct difference in nuclear genome size between the groups. Group 2 also was characterized by a sexual dimorphism in inflorescence structure, which was not observed in Group 1. Interspecific crosses between the groups produced hybrids with drastically reduced pollen fertility and showed that the male is the heterogametic sex (XY) in Group 2, as is the case in *S*. *oleracea* L. (Group 1). Cytogenetic and DNA marker analyses suggested that Group 1 and Group 2 have homomorphic and heteromorphic sex chromosome pairs (XY), respectively, and that the sex chromosome pairs of the two groups evolved from a common ancestral pair. Our data suggest that the *Spinacia* genus may serve as a good model for investigation of evolutionary mechanisms underlying the emergence of heteromorphic sex chromosome pairs from ancestral homomorphic pairs.

Most angiosperms are cosexual (hermaphroditic or monoecious), and dioecy is taxonomically widely dispersed although it is present in only a small percentage of flowering plants (Renner and Ricklefs 1995). The low frequency and wide distribution of dioecy suggest that cosexuality is the ancestral sexual condition, from which dioecy has arisen recently and independently in many evolutionary lineages. The sex of dioecious plants often is determined genetically, and sex chromosome-based sex determination systems have been found in a wide range of angiosperm lineages. Therefore, given the evolutionary history of dioecy, it is plausible that plant sex chromosomes also have evolved recently from autosomes in their cosexual ancestors (Westergaard 1958; Charlesworth 2013).

Heteromorphic sex chromosome pairs have been found in white campion (*Silene latifolia* L.), sorrel (*Rumex acetosa* L.), and hemp (*Cannabis sativa* L.), etc.; however, homomorphic sex chromosome pairs also are found in many dioecious plants, for example, spinach (*Spinacia oleracea* L.), asparagus (*Asparagus officinalis* L.), and papaya (*Carica papaya* L.). Sex chromosome pairs are derived from autosomal pairs, strongly suggesting the evolutionary transition from homomorphic to heteromorphic sex chromosome pairs. Thus, homomorphic and heteromorphic sex chromosomes that have emerged in various angiosperm lineages may represent different evolutionary stages, providing a great opportunity to examine early sex chromosome evolution (Ming *et al.* 2011; Charlesworth 2013).

Spinach (*Spinacia oleracea* L.) is a member of *Chenopodioideae* (a subfamily of the *Amaranthaceae*/*Chenopodiaceae* alliance) (Kadereit *et al.* 2003), and one of the most nutritious leafy vegetables grown worldwide (FAOSTAT, http://faostat3.fao.org/home/index.html). It is commonly considered a dioecious species with wind pollination, although certain cultivars, lines, and crosses can produce individual plants with both male and female flowers (*i.e.*, monoecious plants) (Janick and Stevenson 1955a; Onodera *et al.* 2008). Previous genetic studies, including examinations of various synthetic polyploids, show that spinach possesses a male-heterogametic (XX/XY) sex determination system with an active Y chromosome. Trisomy analysis has located a sex-determining locus on the largest chromosome of the complement (Janick and Stevenson 1954, 1955b; Ellis and Janick 1960; Sugiyama and Suto 1964).

In cytogenetic analyses of a wide range of spinach varieties and germplasm accessions, no obvious heteromorphism of the largest chromosome (sex chromosome, XY) pair has been observed, with rare exceptions (Lorz 1937; Bose and Janick 1961). The largest chromosome has a submedian centromere in most spinach stocks; however, a longer and median (metacentric/homobrachial) variant of the chromosome, which is likely due to a translocation event, was found in a spinach accession (PI 169671) from Turkey (Iizuka and Janick 1962). The longer variant along with the standard chromosome could form a heteromorphic pair.

A recent study using fluorescence *in situ* hybridization (FISH) reported that spinach sex chromosomes might be differentiated by the loss of a 45S rDNA locus from *Y*-bearing members (Lan *et al.* 2006). However, intraspecific copy number variation of ribosomal RNA genes, as well as intraspecific numerical variation of rDNA loci, has been observed in a wide range of plant species (Rogers and Bendich 1987; Kataoka *et al.* 2012), so further verification is required to determine whether variation at the 45S rDNA locus in spinach is associated with sexual dimorphism.

The genus *Spinacia* is known to contain two wild relatives (*S*. *turkestanica* Ilj. and *S*. *tetrandra* Stev.) of cultivated spinach (*S*. *oleracea* L.) (Sneep 1982; Hammer 2001). (Note that *S*. *tetrandra* Roxb. is now regarded as synonymous with *S*. *oleracea* L. [Lorz 1937; Sneep 1982].) Currently, germplasm accessions of wild spinach relatives are stored in gene banks in the United States and Europe and are considered important resources for future spinach breeding. However, little is known about genetic relationships among *Spinacia* species. *S*. *turkestanica* Ilj. has been described as the closest relative of *S*. *oleracea* L. (Hammer 2001), and according to Hooker and Jackson (1895) and Volkens (1893), *S*. *tetrandra* Stev. is distinct from *S*. *oleracea* L. (Lorz 1937). However, recent molecular phylogenetic analyses failed to show that *S*. *tetrandra* Stev. is a distinct species (Hu *et al.* 2007). Lorz (1937) also failed to show any significant karyotypic difference between *S*. *tetrandra* Stev. and *S*. *oleracea* L., and did not observe heteromorphism in their chromosome pairs that could be associated with sex determination.

Interestingly, in contrast to the results of Lorz (1937), Araratjan (1939) found a heteromorphic pair of chromosomes in *S*. *tetrandra* Stev. collected from the vicinity of Yerevan, Armenia. One member of the heteromorphic pair in male plants is 1.75-fold longer than the other, which was equivalent in length to members of a homomorphic chromosome pair in female plants. It is not clear whether the heteromorphism is associated with sex determination. However, Araratjan (1939) indicate that *S*. *tetrandra* Stev. might not only be valuable for future spinach breeding, but also provide new insights into the evolution of plant sex chromosomes.

Because only a few wild spinach accessions were tested in previous studies (Hu *et al.* 2007; Fuentes-Bazan *et al.* 2012), we undertook a molecular phylogenetic analysis of a broad sample of *Spinacia* stocks, including *S*. *turkestanica* Ilj. and *S*. *tetrandra* Stev. accessions that have not yet been analyzed, to facilitate efficient use of wild spinach as breeding materials and establish a research base for sex chromosome evolution in the genus *Spinacia*. Furthermore, to explore evidence for the presence of homomorphic and heteromorphic sex chromosome pairs in the *Spinacia* species, and to verify if the variation at the 45S rDNA locus is associated with sexual dimorphism, we used flow cytometry to estimate nuclear DNA quantity and cytogenetic analysis combined with FISH.

## Materials and Methods

### Plant material

Twenty-one germplasm accessions of three *Spinacia* species (*S*. *oleracea* L., *S*. *turkestanica* Ilj., and *S*. *tetrandra* Stev.) were obtained from the United States Department of Agriculture, the Centre for Genetic Resources, the Netherlands, and the National Institute of Agrobiological Sciences Genebank (Tsukuba, Japan). Three commercial spinach (*S*. *oleracea* L.) cultivars (Mazeran, Nippon, and SPI588) produced by Takii & Co., Ltd. (Kyoto, Japan) and Syngenta Vegetables (Boise, ID), and three spinach breeding lines (86−36, 105−18, and 03−009) produced by Tohoku Seed Co., Ltd. (Utsunomiya, Japan) also were used (Table 1) (Onodera *et al.* 2008). The *Spinacia* plants were grown in a growth chamber (LH-350S; Nippon Medical & Chemical Instruments Co., Ltd, Osaka, Japan) at 20° under an 8-hr photoperiod during the first 4 wk, and subsequently at 20° under a 16-hr photoperiod. Plants bearing more than 20 flower clusters, each of which consisted of 5–16 flowers, were examined to distinguish sexes. Pollen fertility was determined using the Alexander test (Alexander 1969). In total, ∼200 pollen grains collected from an inflorescence with more than five mature flowers (with dehisced anthers) were tested for each plant. Total cellular DNA was prepared from individual plants using the method of Sassa (2007).

**Table 1:** Plant materials used in this study

Species	Accession, Cultivar, or Breeding Line	Germplasm Bank or Breeding Company	Origin*^a^*
*S. oleracea* L.	acc. Ames 26244	USDA	China
	acc. PI 173124	USDA	Turkey
	acc. PI 173972	USDA	India
	acc. PI 181923	USDA	Syria
	acc. PI 217425	USDA	South Korea
	acc. PI 604787	USDA	Afghanistan
	acc. PI 606707	USDA	Netherlands
	acc. JP 25756	NIAS	Japan
	acc. JP 25763	NIAS	Japan
	cv. Mazeran	Takii	
	cv. Nippon	Takii	
	cv. SPI 588	Syngenta	
	bl. 03-009	Tohoku	
	bl. 86-36	Tohoku	
	bl. 105-18	Tohoku	
*S. turkestanica* Ilj.	acc. Ames 23666	USDA	Germany
	acc. PI 494751	USDA	Uzbekistan
	acc. PI 647863	USDA	Turkmenistan
	acc. PI 604792	USDA	Germany
	acc. PI 608713	USDA	Turkmenistan
	acc. CGN 09594	CGN	Uzbekistan
	acc. CGN 09597	CGN	Unknown
*S. tetrandra* Stev.	acc. Ames 23664	USDA	Denmark
	acc. PI 608712	USDA	Germany
	acc. PI 647859	USDA	Georgia
	acc. PI 647860	USDA	Georgia
	acc. PI 647861	USDA	Georgia

acc., germplasm accession; USDA, United States Department of Agriculture; NIAS, National Institute of Agrobiological Sciences; cv., cultivar; bl., breeding line; CGN, Centre for Genetic Resources, the Netherlands.

a Origins are based on the descriptions provided by USDA, CGN, and NIAS.

### Genotyping with sex-linked markers and a derived cleaved amplified polymorphic sequence marker

Spinach male-specific DNAs, T11A and V20A (accession numbers E15132 and E15133; Akamatsu *et al.* 1998), spinach sex chromosome−linked, sequenced-characterized amplified region (SCAR) markers (SP_0007, SP_0008, SP_0016, SP_0017, and SP_0022; Yamamoto *et al.* 2014), and SSR marker SO4 (Khattak *et al.* 2006) were used for genotyping. Amplification reactions and detection of markers were carried out as described previously (Khattak *et al.* 2006; Onodera *et al.* 2008; Onodera *et al.* 2011; Kuwahara *et al.* 2013; Yamamoto *et al.* 2014).

Primers for dCAPS marker SP_0048 (5′-CTTATAGCTGCCATTTAAACACA-3′ and 5′-AACAATCAAGCCTGAACAAAATT-3′) were designed from the spinach *ketohexokinase* (*khk*) gene sequence (approximate nucleotide position 4437–4614 on spinach genome scaffold 60595) (http://bvseq.molgen.mpg.de/Genome/Download/Spinach/; Dohm *et al.* 2014). Amplification reactions for SP_0048 were performed in total volumes of 10 µL containing 10 ng of template DNA, 0.2 µM of each primer, 0.2 mM of each dNTP, and 5 µL of GoTaq Master Mix (Promega Corporation, Madison, WI). Amplification was performed for 30 cycles after initial denaturation at 94° for 2 min. Each cycle consisted of 15 s at 94°, 30 sec at 57° and 1 min at 72°, followed by 5 min at 72°. Before gel electrophoresis analysis, the polymerase chain reaction (PCR) product for SP_0048 was digested with *Dra*I.

### Phylogenetic analysis

Templates for sequencing of five chloroplast intergenic spacers (*trnL-trnF*, *rpl32-trnL*, *trnV-ndhC*, *ndhF-rpl32*, and *psbD-trnT*) and internal transcribed spacer (ITS) regions of nuclear rRNA genes in the *Spinacia* species were generated by PCR using the primer sets shown in Supporting Information, Table S1. Nucleotide sequences of the PCR products were directly determined using the BigDye Terminator v3.1 Cycle Sequencing Kit and Applied Biosystems 3130 Genetic Analyzer (Life Technologies, Carlsbad, CA). The PCRs and sequencing reactions were repeated at least twice to avoid amplification error. The *trnL-trnF* and ITS are used widely for phylogenetic analysis, and the other sequences are highly variable in angiosperms and suited for molecular studies of closely related taxa (White *et al.* 1990; Shaw *et al.* 2007). The chloroplast and nuclear DNA sequences of *Beta procumbens* C. Sm. [accession ID, sp541205-03; NARCH (National Agricultural Research Center for Hokkaido Region), Sapporo, Japan] and *Beta webbiana* Moq. (Syn. *Patellaria webbiana* Moq.) (accession ID, Ames 4515; USDA, NPGS), used as outgroup taxa, also were sequenced. The genus *Beta* is placed in *Betoideae*, which is a small subfamily of the *Amaranthaceae*/*Chenopodiaceae* alliance and is closely related to the *Chenopodioideae* subfamily within the alliance (Kadereit *et al.* 2006). Total cellular DNA from the *Beta* plants was kindly provided by Dr. Tomohiko Kubo (Hokkaido University) (Table S2).

Concatenated chloroplast and nuclear DNA sequences were aligned using MUSCLE software implemented in MEGA 6.0 (Tamura *et al.* 2013). All positions containing gaps and missing data were removed from the dataset (complete deletion option). Phylogenetic trees were constructed with the maximum-likelihood (ML) method using MEGA 6.0. The general time reversible model (Tavaré 1986) was chosen for ML analyses according to the Bayesian Information Criterion using the model test function in MEGA 6.0. Initial tree(s) for the heuristic search were obtained by applying the neighbor-joining method to a matrix of pairwise distances estimated using the maximum composite likelihood approach. The reliability of the ML trees was assessed by 1000 bootstrap replicates.

### Flow cytometric analysis of nuclear DNA

Measurements of relative nuclear DNA amounts were carried out as described in Miyashita *et al.* (2011). A leaf disc (an approximately 5 mm^2^ square) excised from a young leaf of a *Spinacia* plant (from germplasm accessions and cultivars) was used for each measurement. The leaf disc was chopped up in 0.2 mL of nuclei extraction buffer (CyStain UV precise P; Partec, Münster, Germany) together with a sugar beet leaf disc (*Beta vulgaris* strain TK81-MS; Onodera *et al.* 1999), which was used as an internal standard. The suspension of nuclei was filtered through a 30-µm nylon mesh and mixed with 0.8 mL of DAPI solution containing 10 mM Tris, 50 mM sodium citrate, 2 mM MgCl_2_, 1% (w/v) PVP K-30, 0.1% (v/v) Triton X-100, and 2 mg L^−1^ DAPI (pH 7.5). Fluorescent intensity of the nuclei stained with DAPI was measured using a flow cytometer, Partec PA (Partec GmbH, Münster, Germany). The ratio of *Spinacia* plant to sugar beet fluorescent intensity gave the relative nuclear DNA amounts of the examined individuals. A male and a female plant were chosen from each of the examined accessions and cultivars to estimate the relative nuclear DNA amount, and five replicated measurements were conducted for each individual. Significant differences between the mean values of the replicated measurements for each individual were tested by a one-way analysis of variance (ANOVA) with Tukey’s post-hoc test using the R statistical environment (R Development Core Team 2014).

### Karyotyping and FISH

Chromosome observations in mitotic root tips and FISH mapping of rDNA loci (45S and 5S) were carried out as described in Ito *et al.* (2000). Fluorescent microscopy was performed with a Nikon Eclipse E600 (Nikon, Tokyo, Japan). Chromosome images were captured using a CCD camera, CoolSNAP MYO (Photometrics, Tucson, AZ), and analyzed and processed using CHIAS IV (Kato *et al.* 2009), a plugin for ImageJ (http://rsbweb.nih.gov/ij/). The CHIAS IV system was also used to generate color ideograms of metaphase chromosomes based on FISH imaging data: relative chromosome length, position of centromeres, and position and signal intensity of 45S and 5S repeats.

Chromosomes were classified on the basis of their relative arm lengths (long arm/short arm ratios) according to Levan *et al.* (1964). Individual spinach (*S. oleracea* L.) chromosomes were identified with reference to the karyotypic features reported by Ito *et al.* (2000), such as their relative length, the centromeric position, the location of rDNA loci (45S and 5S), and the presence or absence of a satellite. Following Ito *et al.* (2000), we used the nomenclature of spinach chromosomes proposed by Sugiyama and Suto (1964). The present study also referred to the cytogenetic work of Ellis and Janick (1960), who determined that the largest chromosome pair are sex chromosomes in *S. oleracea* L. (Table S3). The sex chromosomes of *S*. *tetrandra* Stev. PI 647859, PI 647860, and PI 647861 were identified based on a sex-associated difference in the nuclear DNA content, as well as on a sex-associated heteromorphism in a chromosome pair.

## Results

### Morphology of *S*. *turkestanica* Ilj. and *S*. *tetrandra* Stev.

Plants were grown from the original seeds (provided by the germplasm banks) of seven accessions of *S*. *turkestanica* Ilj. and five accessions of *S*. *tetrandra* Stev., and were observed to determine their sexual system (hermaphroditism, monoecism, dioecism, etc.) and floral morphology (Table S4). In total, 127 plants from 12 wild *Spinacia* accessions flowered, and both male and female plants were found in each of the accessions, but no monoecious or hermaphrodite plant was observed, suggesting that each accession represents a dioecious population.

Spinach male-specific DNA markers, T11A and V20A (Akamatsu *et al.* 1998; Onodera *et al.* 2008, 2011; Yamamoto *et al.* 2014), amplified in all male plants other than three of the five *S*. *tetrandra* Stev. accessions (PI 647859, PI 647860, and PI 647861) (data not shown). T11A and V20A did not amplify products in any males or females from these three accessions. The results indicate that the same chromosomal region around the male-determining gene(s) may be found in all wild *Spinacia* accessions, other than the three *S*. *tetrandra* Stev. accessions (PI 647859, PI 647860, and PI 647861).

No obvious difference was observed between the appearance of male and female flowers borne on the plants from the 12 wild *Spinacia* accessions and the floral morphology of cultivated spinach (*S*. *oleracea* L.) observed in our previous study (Onodera *et al.* 2008) (Figure S1). However, in the female plants from three accessions of *S*. *tetrandra* Stev. (PI 647859, PI 647860, and PI 647861), female flowers arose from axils not only of upper (bract) leaves, but also of basal leaves (Figure S2). In the other *Spinacia* plants (including male plants from PI 647859, PI 647860, and PI 647861), as well as in spinach (*S*. *oleracea* L.) plants (as illustrated in Figure 1 of Sherry *et al.* [1993]), the male and female flowers arose only from axils of upper (bract) leaves.

**Figure 1:**
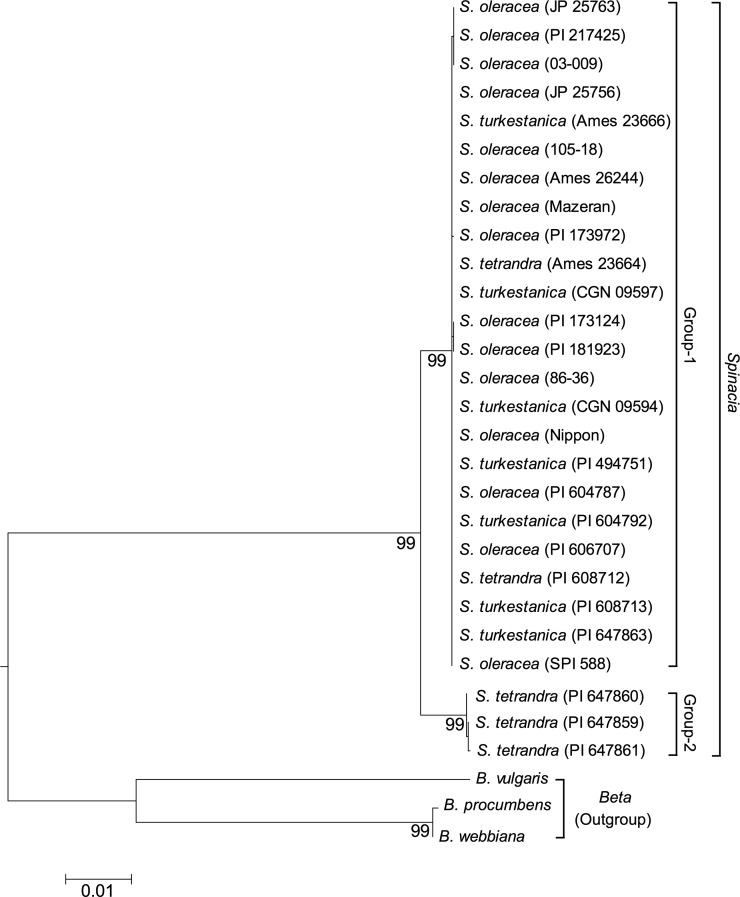
Maximum-likelihood (ML) tree of concatenated DNA sequences consisting of five chloroplast intergenic spacers and nuclear ITS regions from 27 *Spinacia* samples and three outgroup taxa in the genus *Beta*. This tree was constructed using the general time reversible model. Bootstrap percentages (>80%) are listed below branches.

### Phylogenetic analysis of the genus *Spinacia*

Phylogenetic analysis of the three *Spinacia* species was carried out based on concatenated DNA sequences consisting of five chloroplast intergenic spacers and nuclear ITS regions (see Materials and Methods). The aligned data set of 30 concatenated DNA sequences (ranging from 4680 to 5301 bp) composed of 27 ingroup sequences (15 sequences from *S*. *oleracea* L., seven from *S*. *turkestanica* Ilj., and five from *S*. *tetrandra* Stev.) and 3 outgroup sequences from the genus *Beta* (Table S2) included 917 variable sites and 532 parsimony informative sites (79 and 73 within the ingroup, respectively).

As expected, the ML analyses strongly supported the monophyly of the genus *Spinacia* (99% bootstrap support). The ML tree also showed that the genus can be divided into two strongly supported monophyletic groups (99% bootstrap support for each, designated Group 1 and Group 2) (Figure 1). Most of the variation (73 of the 79 variable nucleotides) within the ingroup sequences represented differences between Group 1 and Group 2. Group 1 included all of the 15 *S. oleracea* L. accessions, cultivars, and breeding lines, the seven *S. turkestanica* Ilj. accessions, and two (Ames 23664 and PI 608712) of five *S. tetrandra* Stev. accessions. Group 2 was made up of the remaining three *S*. *tetrandra* Stev. accessions (PI 647859, PI 647860, and PI 647861) (Figure 1).

As mentioned in the preceding section, we observed male-specific amplification of T11A and V20A in Group 1, but no amplification in plants of Group 2. The groups were also distinguished by female flowers in axils of basal leaves; they were observed only in female plants from Group 2, but not from Group 1.

### Analysis of interspecific hybrids between *Spinacia* species

To further characterize the relationships between Group 1 and Group 2, we examined crossability and pollen fertility of hybrids within and between groups. All cross combinations resulted in normal seed setting, and male and female progeny plants that grew normally and flowered (Table 2). Male and female plants from the cross between Group 1 and Group 2 species, and the cross between different Group 1 species bore male and female flowers in axils of upper (bract) leaves, but not in those of basal leaves (data not shown).

**Table 2:** Number of male and female progeny plants from crosses between *Spinacia* species

Female Parent		Male Parent		Progeny Plants			
Group	Species (Accessions/Cultivars)	Group	Species (Accessions/Lines/Cultivars)	Male	Female	Total	Pollen Fertility of Male Progeny Plants
Group 1		Group 1					
	*S*. *turkestanica* Ilj. (Ames 23666)		*S. oleracea* L. (105-18)	2	4	6	>99.0% (*N* = 2)
	*S*. *tetrandra* Stev. (Ames 23664)		*S. oleracea* L. (86-13)	2	5	7	>98.5% (*N* = 2)
	*S*. *tetrandra* Stev. (Ames 23664)		*S*. *turkestanica* Ilj. (Ames 23666)	3	7	10	>98.0% (*N* = 3)
Group 1		Group 2					
	*S. oleracea* L. (Nippon)		*S*. *tetrandra* Stev. (PI 647859)	15	10	25	10.6–19.8% (*N* = 12)
	*S. oleracea* L. (Nippon)		*S*. *tetrandra* Stev. (PI 647861)	6	8	14	10.1–11.6% (*N* = 3)
Group 2		Group 1					
	*S*. *tetrandra* Stev. (PI 647860)		*S. oleracea* L. (Nippon)	2	2	4	16.6–17.9% (*N* = 2)

*N*, number of plants examined.

Furthermore, female spinach (*S. oleracea* L.) plants (XX) pollinated with pollen from Group 2 male plants (*i.e.*, *S*. *tetrandra* Stev. PI 647859 and PI 647861) produced both males and females (Table 2), indicating that males may be the heterogametic sex (XY) in Group 2.

When female plants in Group 2 (*S*. *tetrandra* Stev. PI 647860) were pollinated with pollen from a male plant (XY) from Group 1 (*S. oleracea* L.), all male hybrids were positive for T11A and V20A, but female hybrids were not (data not shown), suggesting that the spinach male-determining factor functions in the genetic background of Group 2.

The within Group 1 male hybrid progeny plants had normal anther dehiscence and high pollen fertility (∼98% stainability in Alexander’s solution; Table 2 and Figure S3). Male intergroup hybrids also exhibited normal anther dehiscence but had drastically reduced pollen fertility (ranging from 10 to 20%), although male plants in the parental accessions and cultivar showed normal pollen fertility (>91%; Table S5).

### Nuclear DNA content in *Spinacia* species

**Figure 2:**
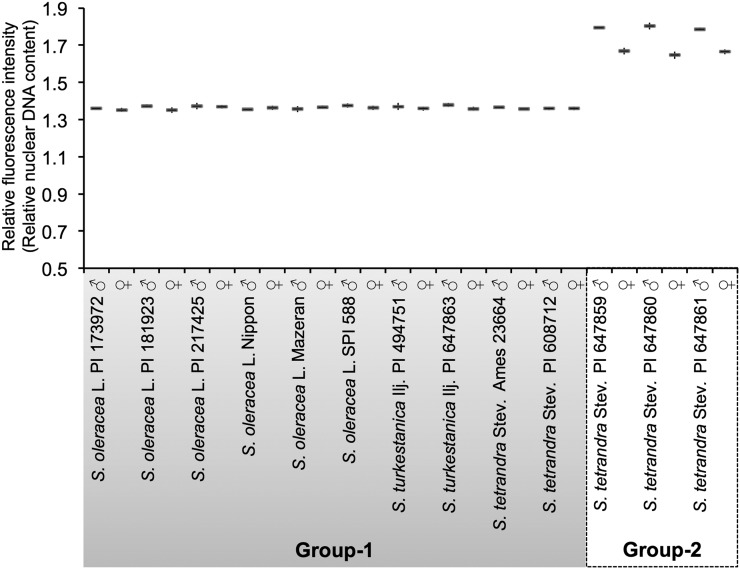
Flow cytometry estimates of relative nuclear DNA amounts in *Spinacia* species. The vertical axis represents relative fluorescence intensity against an internal reference standard, *Beta vulgaris* L. TK81-MS. Error bars represent standard deviations (*N* = 5).

A heteromorphic sex chromosome pair can result in significant differences in the nuclear DNA content between males and females. We measured the DNA content of a single male and female from each of 13 accessions or cultivars from Group 1 and Group 2 using flow cytometry (with five replicated measurements per individual). Relative fluorescence intensity of DAPI-stained nuclei from samples in Group 1 and Group 2 against an internal reference standard (*Beta vulgaris* L. TK81-MS) is summarized in Figure 2. Relative fluorescence intensities were almost invariable (∼1.36) among individuals from the 10 accessions and cultivars classified as Group 1, irrespective of sex, suggesting that there is no obvious size difference between X and Y chromosomes in Group 1 plants.

On the other hand, the relative fluorescence intensity of the plants from the three Group 2 accessions showed a significant difference between males (∼1.79) and females (∼1.66) (one-way ANOVA and post-hoc Tukey’s test, *P* < 0.001; Table S6 and Table S7). Both were significantly larger than the DNA content of plants from Group 1 (one-way ANOVA and post-hoc Tukey’s test, *P* < 0.001; Table S6 and Table S7). As shown in Figure S4, when female plants from Group 1 (*S*. *oleracea* L. SPI 588) and Group 2 (*S*. *tetrandra* Stev. PI 647859) were analyzed simultaneously by flow cytometry, we observed a difference between their genome sizes. We detected distinct fluorescence peaks representing nuclei from the Group 1 and Group 2 female plants. Similarly, when a male and a female plant from Group 2 (*S*. *tetrandra* Stev. PI 647859) were simultaneously analyzed by flow cytometry, we detected distinct fluorescence peaks representing male and female nuclei (Figure S5).

Furthermore, when two or more additional male and female plants from each of the three accessions in Group 2 were examined by a single flow cytometry analysis for each sample, the relative fluorescence intensity of the male and female samples were around 1.79 and 1.66, respectively (data not shown). Taken together, the results support the notion that the sex of Group 2 plants might be determined by a heteromorphic sex chromosome pair, X and Y, with the latter larger than the former.

### Identification of a homomorphic and a heteromorphic sex chromosome pair from Group 1 and Group 2 *Spinacia* plants

**Figure 3:**
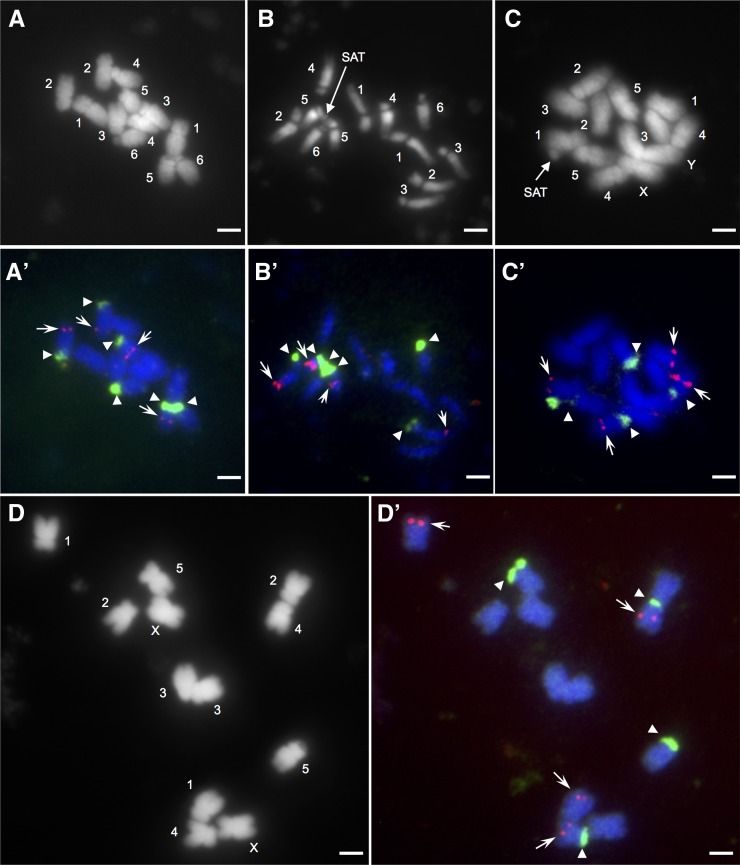
Mitotic metaphase chromosomes in *S. oleracea* L. Mazeran and *S. tetrandra* Stev. PI 647859. (A–D) 4′,6-diamidino-2-phenylindole (DAPI)-stained metaphase chromosomes of *S. oleracea* L. Mazeran (A, male; B, female) and *S. tetrandra* Stev. PI 647859 (C, male; D, female). The Arabic numerals next to chromosomes represent the chromosome number. The sex chromosomes of *S. tetrandra* Stev. are denoted by X and Y. Positions of satellites (SAT) are indicated by arrows. (A′–D′) Fluorescence *in situ* hybridization mapping of 45S (FITC, green) and 5S (Cy3, red) rDNA loci on metaphase chromosomes of *S. oleracea* L. Mazeran (A′, male; B′, female) and *S. tetrandra* Stev. PI 647859 (C′, male; D′, female). Triangles and arrows show the locations of 45S and 5S rDNA loci. Bars = 5 µm.

To confirm the homomorphism of the sex chromosome pair of Group 1 plants indicated by our flow cytometric analysis, we observed mitotic chromosomes in root tip cells prepared from male and female *S. oleracea* L. Mazeran plants. Using microscope observations of metaphase and prometaphase spreads, we confirmed the presence of 2n = 12 chromosomes in both male and female spinach plants (Figure 3 and Figure S6). We did not observe an obvious morphologic difference between members of each chromosomal pair for either sex. We determined the relative length and the arm length ratio of the spinach chromosomes by examining six metaphase spreads from three male and three female plants (Table 3). The chromosome complement of spinach was composed of two pairs of submedian chromosomes and four pairs of subterminal chromosomes, consistent with previous reports (Ellis and Janick 1960; Ito *et al.* 2000). Based on a comparison between our data and that of Ito *et al.* (2000), the chromosomes were numbered 1 through 6 (Table 3 and Table S3). We observed the frequent occurrence of satellites during prometaphase on Chromosomes 5 and 6 (Figure S6 and Table 3). As shown in Figure 3, we also observed a satellite on Chromosome 5 in a metaphase spread. These results agree with Sugiyama and Suto (1964), though other studies have reported a satellite on Chromosome 5 but not on Chromosome 6 (Ellis and Janick 1960; Ito *et al.* 2000) (Table S3).

**Table 3:** A comparison of the karyotypic characteristics of *S. oleracea* L. Mazeran and *S*. *tetrandra* Stev. PI 647859

Chromosome No.	Centromere Position	Relative Length (%)	Arm Ratio*^a^*	45S*^b^*	5S*^b^*	Satellite*^b^*
*S. oleracea* L. Mazeran						
1	Submedian	19.6	1.73			
2	Submedian	18.2	2.17	S	L	
3	Subterminal	17.3	3.44			
4	Subterminal	16.0	3.36			
5	Subterminal	14.5	3.11	S	L	S
6	Subterminal	14.4	6.61	S		S
Total length		100				
*S*. *tetrandra* Stev. PI 647859						
X	Median	18.1	1.29			
Y	Median	29.9	1.12			
1	Submedian	16.7	2.39		L	
2	Submedian	16.6	2.84			
3	Submedian	14.8	1.85			
4	Submedian	17.9	1.86	S	L	S
5	Submedian	15.9	2.46	S		S
Total length (X)		100				
Total length (Y)		111.8				

Autosomes of *S. tetrandra* Stev. PI 647859 were ordered according to their similarity in the possession of rDNA loci (45S and 5S) to those of *S. oleracea* L. Mazeran.

a r-index (long/short arm length ratio; Levan *et al.* 1964).

b Positions of the rDNA loci (45S and 5S) and the satellites on the short (S) or long (L) arm.

To re-examine the previous finding of Lan *et al.* (2006) that heteromorphism is marked by a presence or absence difference of a 45S rDNA site between the spinach sex chromosomes, the metaphase spreads were hybridized *in situ* with a 45S rDNA probe and a 5S rDNA probe. Using FISH, we detected three pairs of 45S rDNA signals, on the short arms of Chromosomes 2, 5, and 6 [including a weak 45S rDNA signal on Chromosome 2, consistent with Ito *et al.* (2000)], and two pairs of 5S rDNA signals on the long arms of Chromosomes 2 and 5 (Figure 3 and Table 3). However, contrary to the results of Lan *et al.* (2006), we did not detect a chromosome pair that differed by the presence or absence of a 45S rDNA site.

Chromosomes 2, 5, and 6, have not been associated with sex determination. The sex-determining locus has been placed on Chromosome 1 (see Table S3) (Ellis and Janick 1960). The heteromorphic pair identified by Lan *et al.* (2006) seems to correspond to Chromosome 2 (the second longest pair) rather than Chromosome 1 (the longest pair), based on the images shown in their report. Interestingly, Iizuka and Janick (1962) found a variant of Chromosome 2 (synonymous with Chromosome 3 in Ellis and Janick [1960]), which can be distinguished from the standard form by a satellite on the short arm, and demonstrated that the variation is not associated with sex. Given that the 45S rDNA repeat is associated with formation of a secondary constriction and satellite, Lan *et al.* (2006) may have observed variation of the 45S rDNA locus on Chromosome 2.

We also observed chromosomes in root tip cells prepared from male and female *S. tetrandra* Stev. PI 647859 plants (Group 2). As shown in Figure 3 and Figure S6, the metaphase and prometaphase observations confirmed the presence of 2n = 12 chromosomes in both male and female plants. We determined the relative length and the arm length ratio of the spinach chromosomes by examining six metaphase spreads prepared from three male and three female plants. As shown in Table 3, the chromosome complement of *S. tetrandra* Stev. PI 647859 was composed of one pair of median chromosomes and five pairs of submedian chromosomes, suggesting that Group 2 plants have a different karyotype from that of Group 1. Furthermore, no obvious morphologic differences were found between the chromosomal pairs in the female *S. tetrandra* Stev. PI 647859 plants. In contrast, in the male plants, one of the six pairs consisted of unequal median chromosomes: one was approximately 1.65 times longer than the other, which corresponded to the largest chromosome in the complement of a female plant.

The members of the remaining five submedian pairs were of equal length in both sexes (Figures 3 and Figure S6, and Table 3). These five chromosome pairs can be readily classified into three types according to their size, as follows: a pair of large chromosomes (relative length, ∼17.9%) (Table 3), two pairs of middle-sized chromosomes (relative length, ∼16.6–16.7%), and two pairs of small chromosomes (relative length, ∼14.8–15.9%). To further discriminate between the five pairs of submedian chromosomes, we physically mapped 45S rDNA and 5S rDNA to mitotic metaphase chromosomes. As shown in Figure 3, we detected two pairs of signals for each of the 45S and 5S rDNA repeats in a male and a female metaphase spread. When comparing chromosome idiograms of *S. tetrandra* Stev. PI 647859 and *S. oleracea* L. Mazeran using the FISH mapping data (Figure S7), we found a similar distribution of rDNA loci. Therefore, we assigned chromosome numbers in *S*. *tetrandra* Stev. PI 647859 (Group 2) based on similar *S*. *oleracea* L. (Group 1) chromosomes.

As shown in Figures 3 and Figure S7 and summarized in Table 3, the large and small members of the median pair were identified as Y and X, respectively. The large submedian pair carrying both 45S and 5S rDNA repeats was designated as Chromosome 4. The middle submedian pairs with and without the 5S rDNA repeats were named Chromosomes 1 and 2, respectively. The small submedian pairs with and without 45S rDNA were numbered 5 and 3, respectively.

It should be noted that Chromosomes 4 and 5 of *S. tetrandra* Stev. (Group 2) were characterized by the occurrence of a satellite on their short arms, which was also true of Chromosomes 5 and 6 of *S*. *oleracea* L. (Group 1) (Figure 3, Figure S6, and Table 3).

### Identification of homeology between the sex chromosomes in Group 1 and Group 2 plants

To verify that the sex chromosomes in Group 1 and Group 2 plants were derived from a common ancestor, we tested an SSR marker (SO4) and five SCAR markers (see the section *Materials and Methods*) linked to the sex chromosomes in *S. oleracea* L. (Onodera *et al.* 2011; Yamamoto *et al.* 2014) in 20 male and 17 female plants from original seeds of the three Group 2 accessions (PI 647859, PI 647860, and PI 647861). It is worth mentioning that the nucleotide sequences of two of the five SCAR marker loci (SP_0008 and SP_0016) showed perfect homology to sequences spanning exon-intron junctions of the *chloride channel protein* (*clc-e*) and *ketohexokinase* (*khk*) genes on spinach genome scaffolds 65004 and 60595 (Dohm *et al.* 2014), respectively. The sequences of the remaining three SCAR marker loci did not have any homology to known protein-coding gene regions (data not shown).

**Figure 4:**
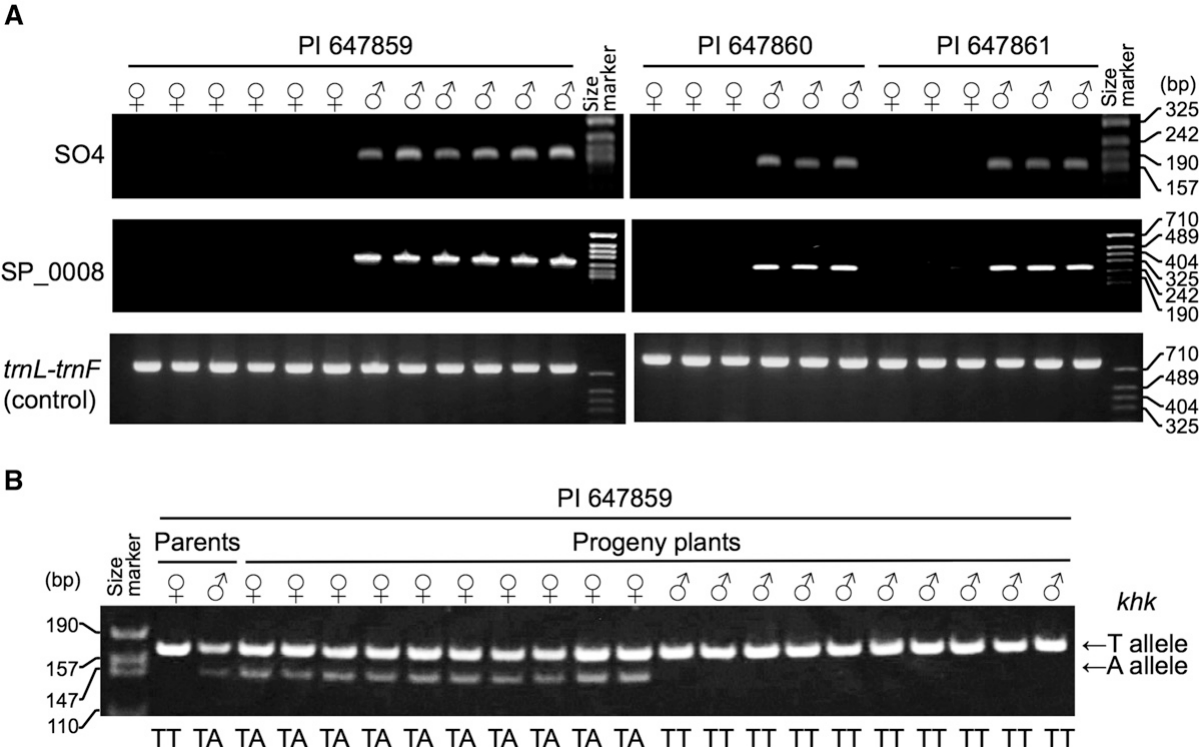
Polymerase chain reaction−based genotyping of male and female plants from *S*. *tetrandra* Stev. PI 647859, PI 647860, and PI 647861. (A) Genotyping of microsatellite SO4 and sequenced-characterized amplified region (SCAR) marker SP_0008. (B) Single-nucleotide polymorphism typing for the *ketohexokinase* (*khk*) locus in parental and progeny plants of a cross between a male plant and a female plant in PI 647859, using dCAPS marker SP_0048. DNA bands representing the “T” and “A” alleles are indicated by arrows. SNP genotypes (TT or TA) at the *khk* locus are shown below the gel image.

As shown in Table 4 and Figure 4, SO4 and SCAR marker SP_0008 amplified in all male plants from Group 2 but not in females. The alleles for SO4 and SP_0008 carried by the males were named *SO4*^12^ and *SP_0008*^277^ based on the number of SSR repeat motifs and the molecular weight (bp), respectively. The same result was obtained for the progeny of intra-accession crosses using PI 647859 and PI 647861 (Table 4). These results indicated that *SO4*^12^ and *SP_0008*^277^ are linked to the sex-determining factor and located on the Y chromosome in Group 2 plants, and that the female plants are homozygous for null alleles (*SO4*^null^ and *SP_0008*^null^) of SO4 and SP_0008, whereas the male plants are heterozygous at the loci. On the other hand, the remaining four SCAR markers failed to amplify any product from males or females in Group 2.

**Table 4:** Genotypes at the SO4 and SP_0008 loci in male and female plants from Group 2

*S. tetrandra* Stev.	Male		Female		
	*SO4*^12^*SO4*^null^ *SP_0008*^277^*SP_0008*^null^	*SO4*^null^*SO4*^null^ *0008*^null^*0008*^null^	*SO4*^12^*SO4*^null^ *SP_0008*^277^*SP_0008*^null^	*SO4*^null^*SO4*^null^ *0008*^null^*0008*^null^	Total
PI 647859	11	0	0	7	18
PI 647859*^a^*	10	0	0	23	33
PI 647860	3	0	0	6	9
PI 647861	6	0	0	4	10
PI 647861*^a^*	2	0	0	5	7
Total	32	0	0	45	77

a Progeny families from a cross between a male plant and a female plant in PI 647859 or PI 647861.

To further verify the homeology between the sex chromosomes in Group 1 and Group 2 plants, a dCAPS marker was developed to detect a single nucleotide polymorphism (TT and TA genotypes) at the *khk* locus in maternal and paternal plants of the progeny families from the cross between a male plant and a female plant in PI 647859. As presented in Figure 4, the maternal and paternal plants had TT and TA genotypes, respectively. The dCAPS marker analysis revealed that all females (*N* = 23) in the families had the TA genotype, and all males (*N* = 10) had the TT genotype (Figure 4 and Table S8). The results indicate that the “A” and “T” alleles in the paternal plant are X-linked and Y-linked, respectively. Taken together, our results suggest that SO4, SP_0008 (*clc-e*), and the *khk* gene locate on sex chromosomes in both Group 1 and Group 2 plants.

## Discussion

Our molecular phylogenetic analysis revealed that *Spinacia* species can be classified into two monophyletic groups (Group 1 and 2), which is consistent with the observed differences in the genome size and karyotype. Previous studies (Hu *et al.* 2007; Fuentes-Bazan *et al.* 2012) also have analyzed germplasm accessions (Ames 23666, Ames 23664, and PI 608712) of the wild species (*S. turkestanica* Ilj. and *S. tetrandra* Stev.); however, these were assigned to Group 1 along with all examined *S. oleracea* L. samples in our analysis. This may explain the failure of previous studies to identify phylogenetic divergence within the genus.

Based on the view of relationships among the three *Spinacia* species described by Hammer (2001) and Lorz (1937) (see the *Introduction*), the wild *Spinacia* germplasm accessions, which were classified together with *S*. *oleracea* L. as Group 1, should be assigned to *S. turkestanica* Ilj. We will therefore treat Ames 23664 and PI 608712 as *S*. *turkestanica* Ilj., not as *S. tetrandra* Stev. Spinach (*S*. *oleracea* L.) shows a wide range of variation in morphological characters such as leaf blade shape, length and color of petiole (http://www.ars-grin.gov/∼dbmuqs/cgi-bin/export.pl?action=dnospread&eno=492382&format=html), and fruit shape (Sneep 1982). Consequently, at least in our experiments, it is difficult to discriminate among *Spinacia* plants based on their morphology. In fact, presence or absence of flowers at the axils of basal leaves in female plants was the only obvious morphological difference between Group 1 (*S*. *oleracea* L. and *S*. *turkestanica* Ilj.) and Group 2 (*S*. *tetrandra* Stev.) that we observed. This lack of apparent differences might result in misclassification of *Spinacia* germplasm accessions. The variant sites in chloroplast intergenic spacers and nuclear ITS could provide ideal markers for the curation of the *Spinacia* germplasm collection.

As mentioned in the *Introduction*, Araratjan (1939) found a heteromorphic pair of median chromosomes in *S*. *tetrandra* Stev. collected from Armenia. One member of the heteromorphic pair in male plants is 1.75-fold longer than the other, similar to our estimated X:Y length ratio (∼1.65). Considering that the *S*. *tetrandra* Stev. accessions PI 647859, PI 647860, and PI 647861 originated in Georgia (a neighboring country of Armenia), the “*S*. *tetrandra* Stev.” plants examined by Araratjan (1939) might be assigned to Group 2. On the other hand, our findings differ from those of Lorz (1937), who found no cytological evidence to support the classification of *S*. *oleracea* L. and *S*. *tetrandra* Stev. as distinct species. It is possible that he examined “wild spinach” in Group 1 (*e.g.*, *S*. *turkestanica* Ilj.) rather than “*bona fide*” *S*. *tetrandra* Stev.

The genome size (C-value) varies approximately 1000-fold among angiosperms (Plant DNA C-values Database; http://data.kew.org/cvalues/). Although most of the variation resides at greater taxonomic levels, significant differences in genome size are found among species within families and even within genera, suggesting that genome size diversification is an important process during speciation in plants (Greilhuber 1998). In this context, it is possible that the genome size difference between Group 1 and Group 2 plants reflects the speciation process in the genus *Spinacia*.

The difference between the karyotypes of the two groups cannot be explained only by a difference in the sex chromosomes (*i.e.*, homomorphic/heteromorphic pairs and submedian/median chromosomes), but is due to morphological variation (*i.e.*, centromeric position or arm ratio) across most autosomes. However, our cytogenetic analysis does not specifically support existence of extensive chromosomal rearrangements (*e.g.*, translocation) during differentiation of the two monophyletic lineages in *Spinacia*. Furthermore, unlike the variant of the spinach sex chromosome reported by Iizuka and Janick (1962, 1963), the heteromorphic sex chromosome pair in Group 2 is unlikely to be due to reciprocal translocation between an autosome and the sex chromosome, because male plants in Group 2 showed normal pollen fertility. If such a translocation was involved, fertility would be reduced by the irregular and unequal distribution of chromosomes involved in the translocation at anaphase I of meiosis, as previously reported (Iizuka and Janick 1962, 1963). Therefore, it seems likely that the reduced fertility of the hybrid plants between Group 1 and Group 2 was due to neither the difference in the sex chromosomes nor chromosome rearrangements, although the latter is reportedly responsible for hybrid sterility in *Helianthus* (Rieseberg *et al.* 1995). Male and female sterility can be caused by a genetic interaction between incompatible alleles brought together in the hybrid progeny, as well as between incompatible genes at different loci (Rieseberg and Blackman 2010; Ouyang *et al.* 2010). Such genetic incompatibility might be involved in the pollen abortion of hybrid plants between Group 1 and Group 2. Nevertheless, more work is necessary to test this hypothesis further.

Previous linkage studies showed widespread recombination in the spinach sex chromosomes except in the vicinity of the male-determining factor (*Y*) (Khattak *et al.* 2006; Onodera *et al.* 2011; Yamamoto *et al.* 2014), consistent with little or no morphological differentiation of the sex chromosomal pair in Group 1. The homomorphic and heteromorphic sex chromosome pairs in Group 1 and Group 2 most likely evolved from a common ancestral sex chromosome pair because their sex-determining genes were linked to common genes. The genus *Silene* includes species groups with sex chromosomes that evolved independently from different autosomal pairs (Mrackova *et al.* 2008), whereas the *Spinacia* homomorphic and heteromorphic sex chromosomes are thought to be homologous.

In plants with heteromorphic sex chromosome systems (*e.g.*, *Silene latifoli* and *Rumex acetosa*), the Y chromosomes are often larger than their X homologs containing extensive nonrecombining regions (MSY, Male-specific region of Y) rich in repetitive elements (Charlesworth 2013). Furthermore, recent studies showed that the papaya Y chromosome has a small nonrecombining region, which is more than twice as large and contains more repetitive sequences (including transposable elements) than the corresponding region of the X chromosome; the X and Y chromosomes are cytologically indistinguishable but are heteromorphic at the molecular level (Wang *et al.* 2012; Aryal and Ming 2014; VanBuren *et al.* 2015). Theoretical and experimental evidence suggests that the evolution of heteromorphic sex chromosomes involves the suppression of recombination in a small region containing multiple sex-determining factors [*e.g.*, male (stamen)-promoting factors and female (gynoecium) suppressors] on a proto-Y chromosome that is homologous to its counterpart (proto-X), and subsequent expansion of the MSY associated with mutation accumulation (*e.g.*, accumulation of transposable elements and repeated sequences) (Bergero and Charlesworth 2009; Charlesworth 2013). These findings indicate that heteromorphism can evolve without chromosomal translocations, are consistent with the accumulation of transposable elements and repeated sequences in nonrecombining regions being responsible for expansion of MSY, and may explain why plant Y chromosomes tend to be larger than their X counterparts. Although the three DNA markers cosegregated perfectly with sex in our small segregating population of *S*. *tetrandra* Stev. further marker-based linkage analysis is needed to test whether recombination is suppressed across a large region of the *S*. *tetrandra* Stev. Y.

Evolutionary theory predicts the accumulation of sexually antagonistic mutations on nascent sex chromosomes and subsequent cessation of recombination between the sexually antagonistic locus and the sex-determining locus, leading to MSY expansions (Bergero and Charlesworth 2009; Bachtrog 2013). The accumulation of sexually antagonistic mutations could be correlated with greater sexual dimorphism (Charlesworth and Mank 2010); in this context, the sex-specific inflorescence structure in *S. tetrandra* Stev., which was not observed in *S. oleracea* L. or *S. turkestanica* Ilj., may be related to the evolution of heteromorphic sex chromosomes and the apparent expansion of the MSY in *S*. *tetrandra* Stev. On the basis of the male heterogamety in this species, flower formation in basal leaves’ axils is probably inherited as a sex-linked recessive trait. Given that the alleles at the trait locus have sexually antagonistic effects, it seems reasonable to assume that this locus may have contributed the evolution of a nonrecombining region and heteromorphism of the sex chromosomes in *Spinacia*. Nevertheless, more studies must be conducted to test this hypothesis.

## Supplementary Material

Supporting Information
