# Supplementary material for: Evidence for a Common Origin of Homomorphic and Heteromorphic Sex Chromosomes in Distinct Spinacia Species
Source: G3 (Bethesda). 2015 Jun 5;5(8):1663–73. doi: 10.1534/g3.115.018671 (PMC4528323; doi:10.1534/g3.115.018671)
Supplement: Supporting Information [file supp_5_8_1663__index.html]

Evidence for a Common Origin of Homomorphic and Heteromorphic Sex Chromosomes in Distinct Spinacia Species — Supporting Information 

# Evidence for a Common Origin of Homomorphic and Heteromorphic Sex Chromosomes in Distinct *Spinacia* Species

## Supporting Information for Fujito *et al.*, 2015

**Files in this Data Supplement:**

- Supporting Information - Figures S1-S7 and Tables S1-S8 (PDF, 970 KB)
- Figure S1 - Floral morphology of *S. turkestanica* Ilj. and *S. tetrandra* Stev. (PDF, 170 KB)
- Figure S2 - Axils of basal leaves of a male plant and female individuals from *S. tetrandra* Stev. PI 647861. (PDF, 212 KB)
- Figure S3 - Pollen fertility of interspecific hybrids between *Spinacia* species. (PDF, 119 KB)
- Figure S4 - A histogram of relative DNA amount obtained after the flow cytometric analysis of nuclei isolated from female plants of *S. oleracea* L. SPI 588 and *S. tetrandra* Stev. PI 647861. (PDF, 116 KB)
- Figure S5 - A histogram showing the nuclear DNA amount in a single plant of *B. vulgaris* L. TK81-MS, and a single male and female of *S. tetrandra* Stev. PI 647859. (PDF, 116 KB)
- Figure S6 - DAPI-stained mitotic prometaphase chromosomes in *S. oleracea* L. Mazeran and *S. tetrandra* Stev. PI 647859. (PDF, 170 KB)
- Figure S7 - Color idiograms illustrating the metaphase chromosome complements of *S. oleracea* L. Mazeran and *S. tetrandra* Stev. PI 647859. (PDF, 102 KB)
- Table S1 - Primer sequences used to amplify and sequence chloroplast intergenic spacers and internal transcribed spacer (ITS) regions of nuclear rRNA genes. (PDF, 62 KB)
- Table S2 - Nucleotide sequences used for the phylogenetic analysis. (PDF, 75 KB)
- Table S3 - Chromosome designation in spinach. (PDF, 61 KB)
- Table S4 - Observed number of males and females from germplasm accessions of the wild *Spinacia* species. (PDF, 64 KB)
- Table S5 - Pollen fertility of male plants in the parental spinach cultivar and *S. tetrandra* Stev. accessions of inter-group hybrids. (PDF, 55 KB)
- Table S6 - One-way ANOVA for the nuclear DNA amounts of 26 *Spinacia* plants presented in Figure 2. (PDF, 63 KB)
- Table S7 - Post hoc Tukey's multiple comparisons of the nuclear DNA amounts between individuals. (PDF, 81 KB)
- Table S8 - Single nucleotide polymorphism typing for the *ketohexokinase* (*khk*) locus in progeny plants from the cross between a male and a female plant in PI 647859, using dCAPS marker SP\_0048. (PDF, 57 KB)
