# Supplementary material for: Evidence for a Common Origin of Homomorphic and Heteromorphic Sex Chromosomes in Distinct Spinacia Species
Source: G3 (Bethesda). 2015 Jun 5;5(8):1663–73. doi: 10.1534/g3.115.018671 (PMC4528323; doi:10.1534/g3.115.018671)
Supplement: Supporting Information [file supp_g3.115.018671_018671SI.pdf]

G3: Genes | Genomes | Genetics

Evidence for a common origin of homomorphic and heteromorphic sex chromosomes in distinct *Spinacia* species

Satoshi Fujito[*, 1], Satoshi Takahata[*, 1], Reimi Suzuki[*, 1], Yoichiro Hoshino[§], Nobuko Ohmido[†], Yasuyuki Onodera[*, 2]

[*]Research Faculty of Agriculture, Hokkaido University, N-9, W-9, Sapporo 060-8589, Japan

[§]Field Science Center for Northern Biosphere, Hokkaido University, N-11, W-10, Sapporo 060-0811, Japan

[†]Graduate School of Human Development and Environment, Kobe University, Kobe 657-8501, Japan

[1]These authors contributed equally to this study.

[2]Corresponding author

The nucleotide sequences determined in this study have been deposited at DNA Data Bank of Japan (DDBJ) (accession numbers: AB935607–AB935634, AB935636–AB935689, AB935691, LC009789–LC009816, LC009818–LC009846, LC009848–LC009876, and LC009878).

DOI: 10.1534/g3.115.018671

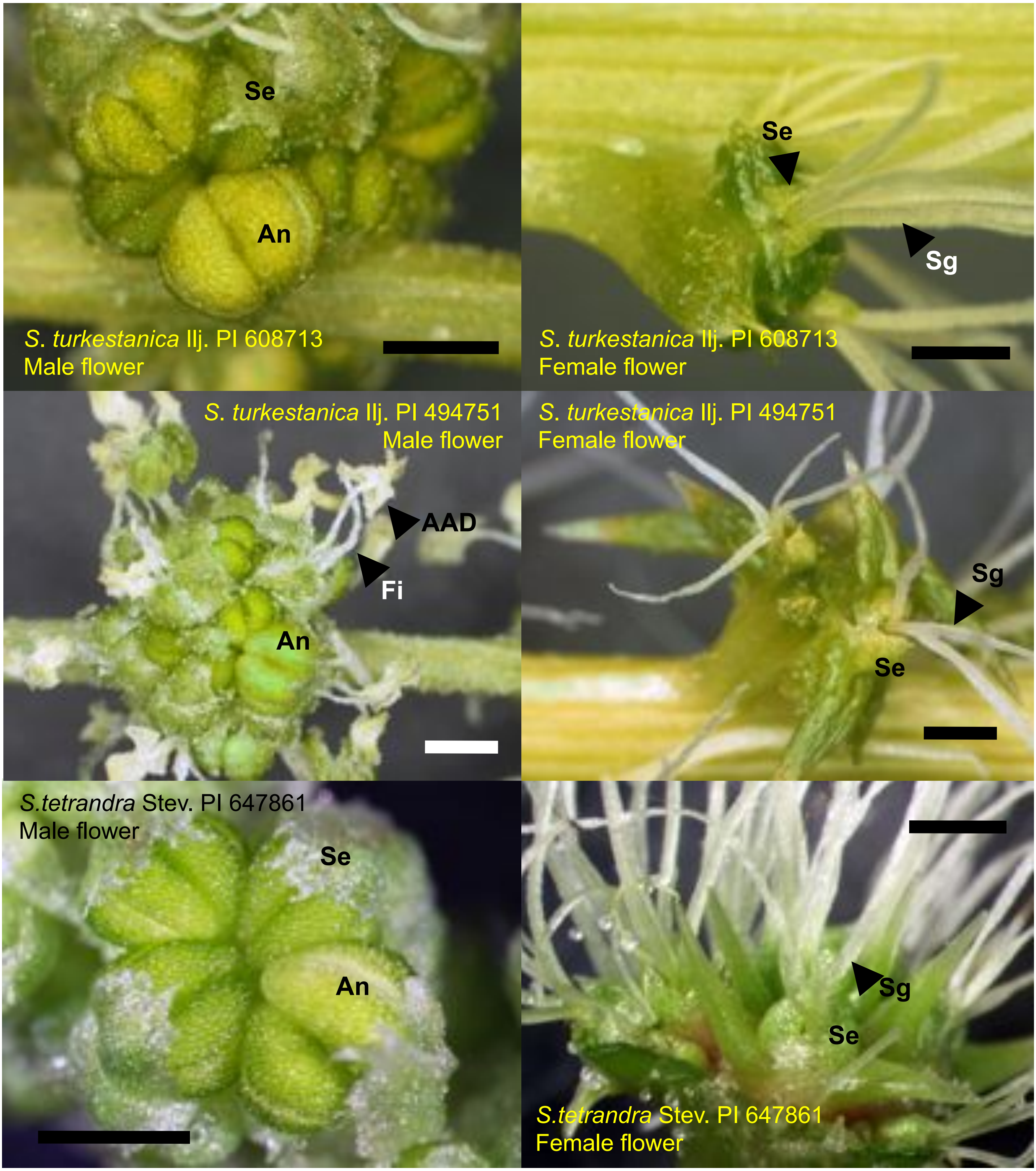

Figure S1. Floral morphology of *S. turkestanica* Ilj. and *S. tetrandra* Stev. Se, sepal; An, anther; AAD, anther after dehiscence; Fi, filament; Sg, stigma. Bar = 1.0 mm

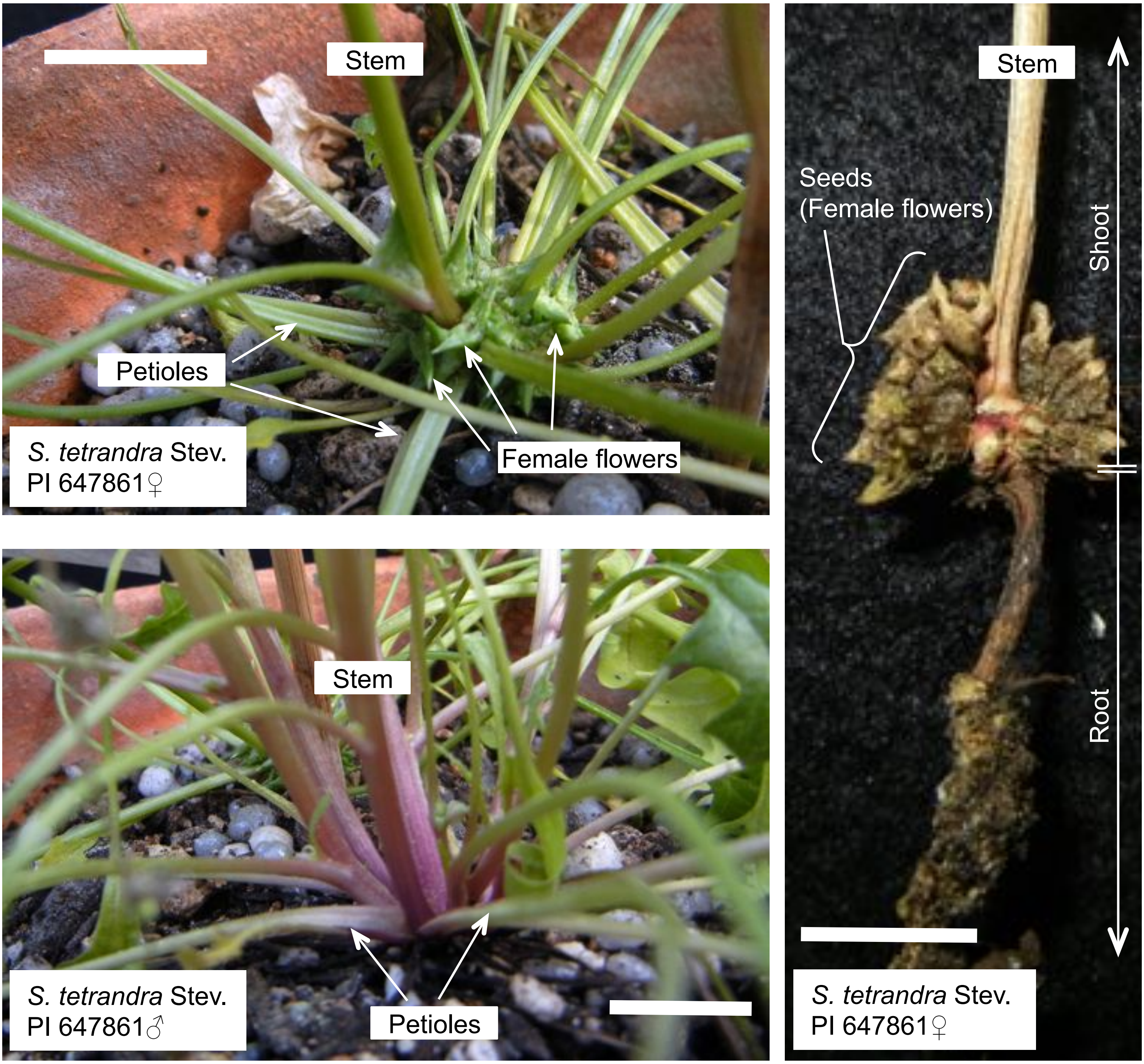

Figure S2. Axils of basal leaves of a male plant and female individuals from *S. tetrandra* Stev. PI 647861. Please note that flowers arose only from axils at base of a shoot of a female plant. Bar = 1.0 cm

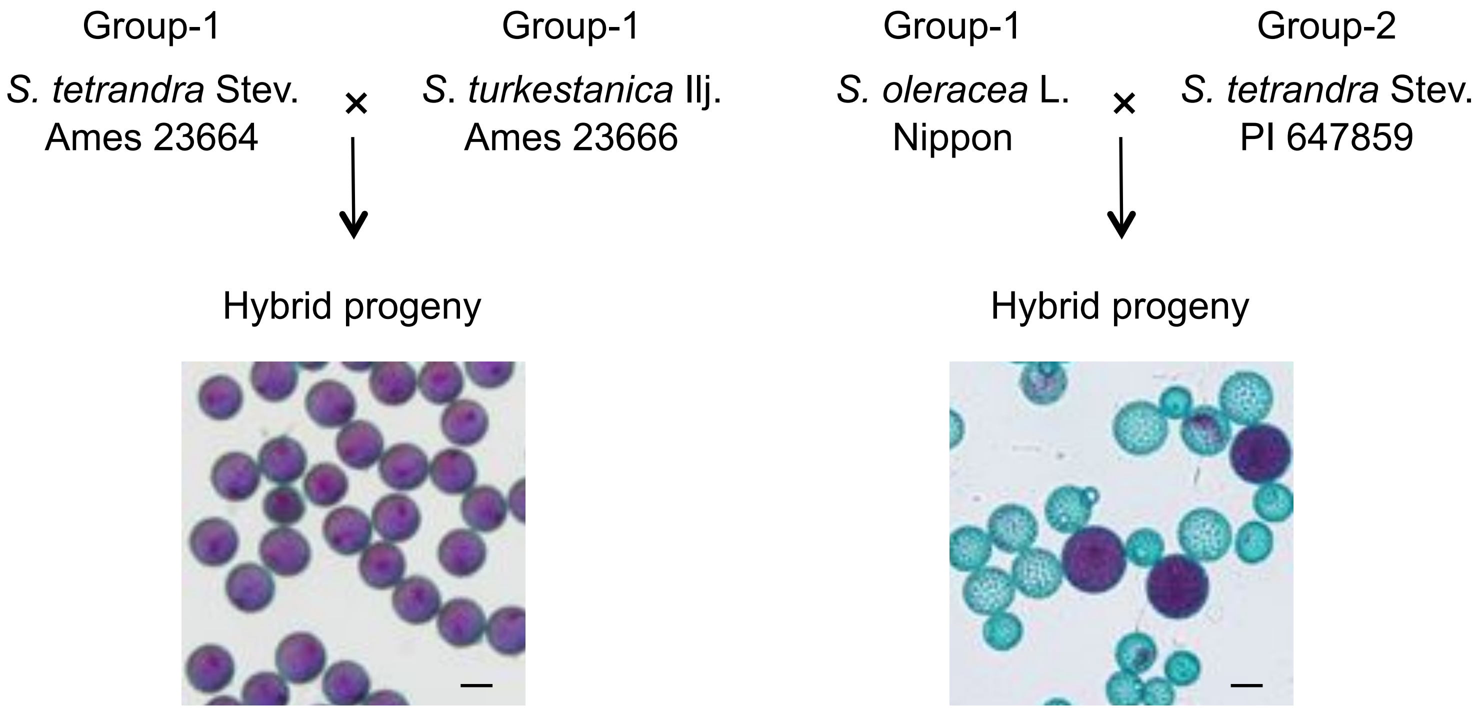

Figure S3. Pollen fertility of interspecific hybrids between *Spinacia* species. Pollen grains were stained with Alexander solution. Viable and non-viable pollen grains stained purple and pale-blue-green, respectively. Bars = 20 µm.

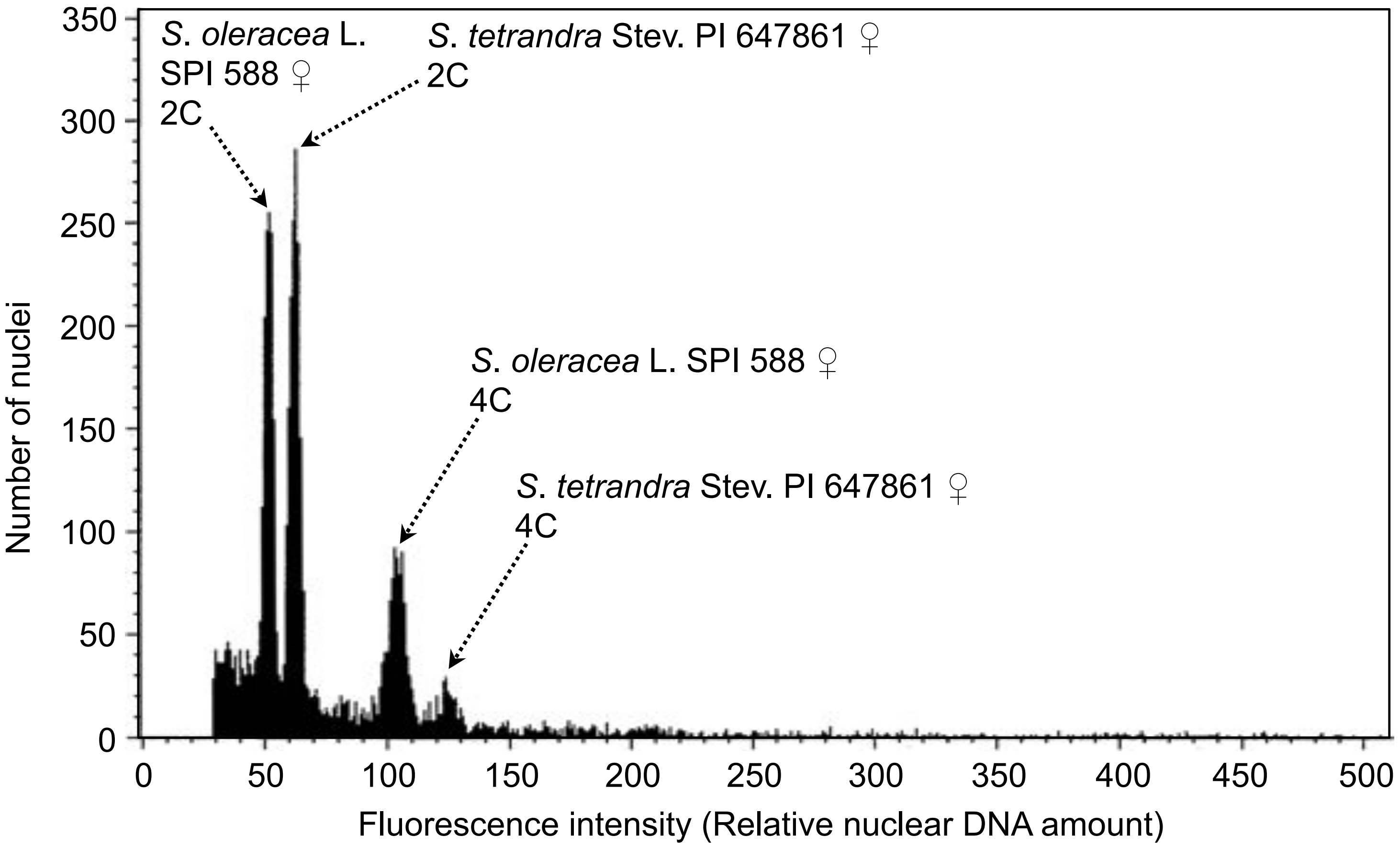

Figure S4. A histogram of relative DNA amount obtained after the flow cytometric analysis of nuclei isolated from female plants of *S. oleracea* L. SPI 588 and *S. tetrandra* Stev. PI 647861. Arrows indicate 2C peaks (nuclei of cells in the G0/G1 phase) and 4C peaks (G2/M phase).

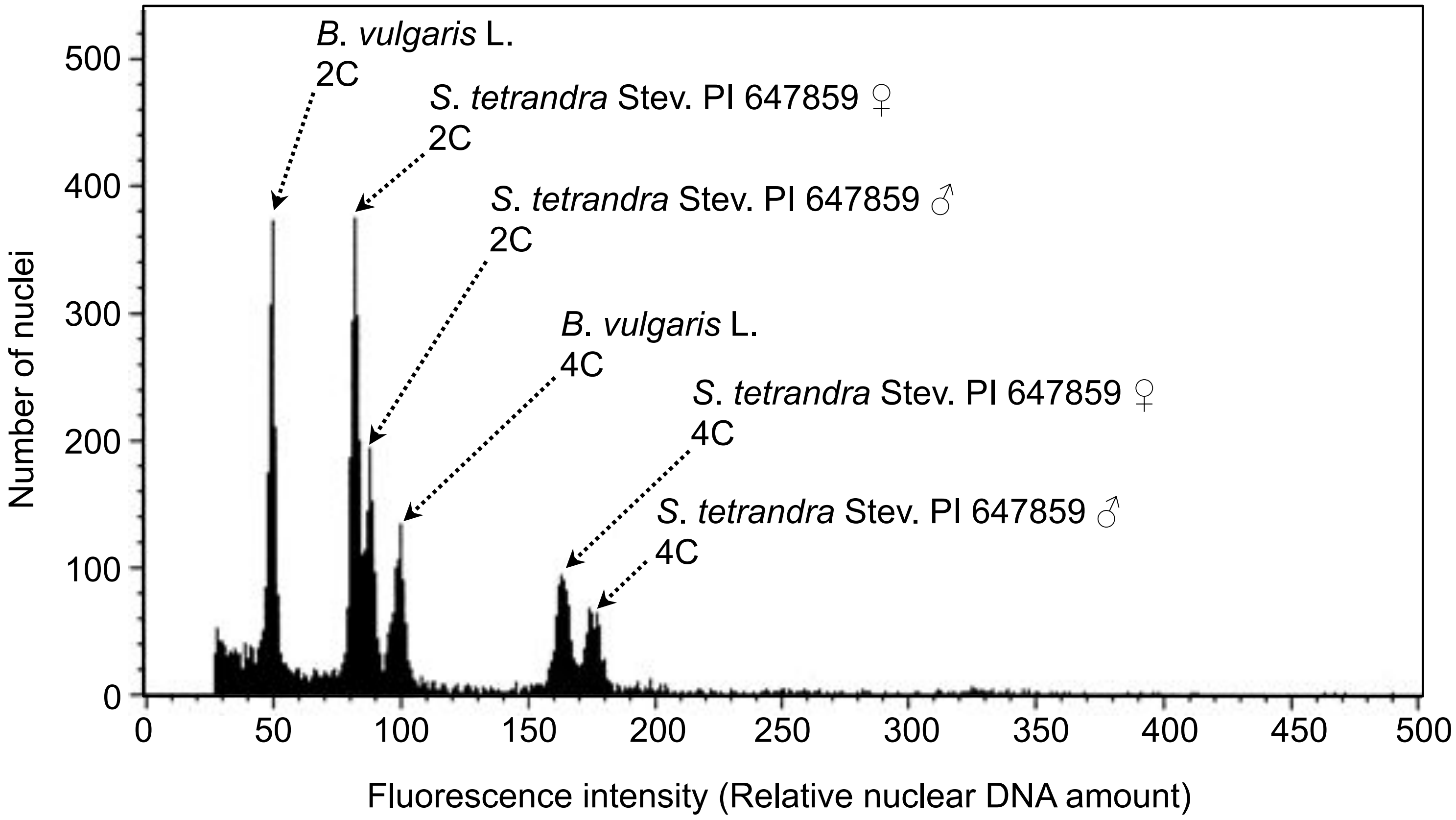

Figure S5. A histogram showing the nuclear DNA amount in a single plant of *B. vulgaris* L. TK81-MS, and a single male and female of *S. tetrandra* Stev. PI 647859. Arrows indicate 2C peaks (G0/G1 phase) and 4C peaks (G2/M phase).

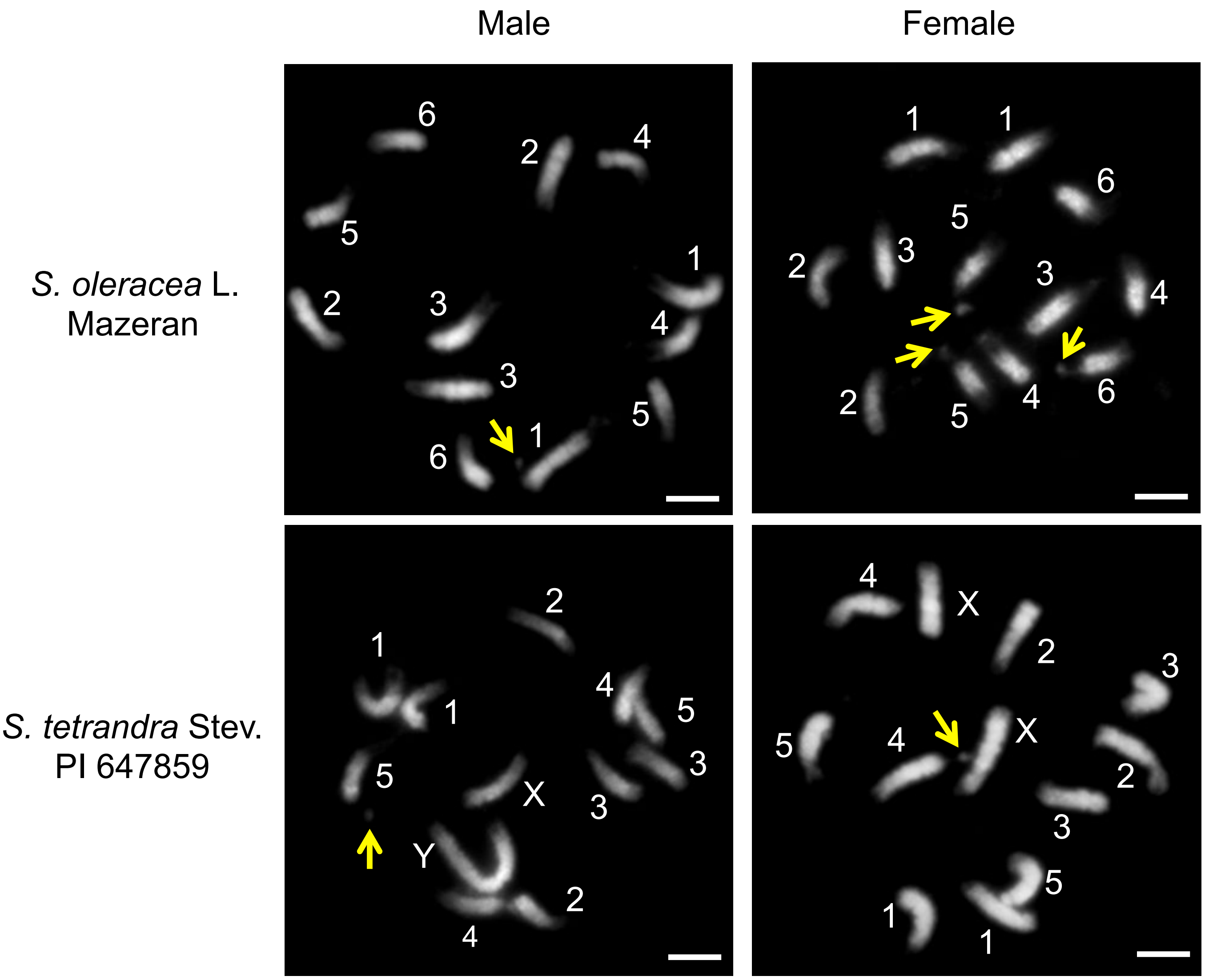

Figure S6. DAPI-stained mitotic prometaphase chromosomes in *S. oleracea* L. Mazeran and *S. tetrandra* Stev. PI 647859. Note that a heteromorphic chromosome pair (XY) can be found only in a male of *S. tetrandra* Stev. PI 647859. Arrows indicate satellites. Bars = 5µm.

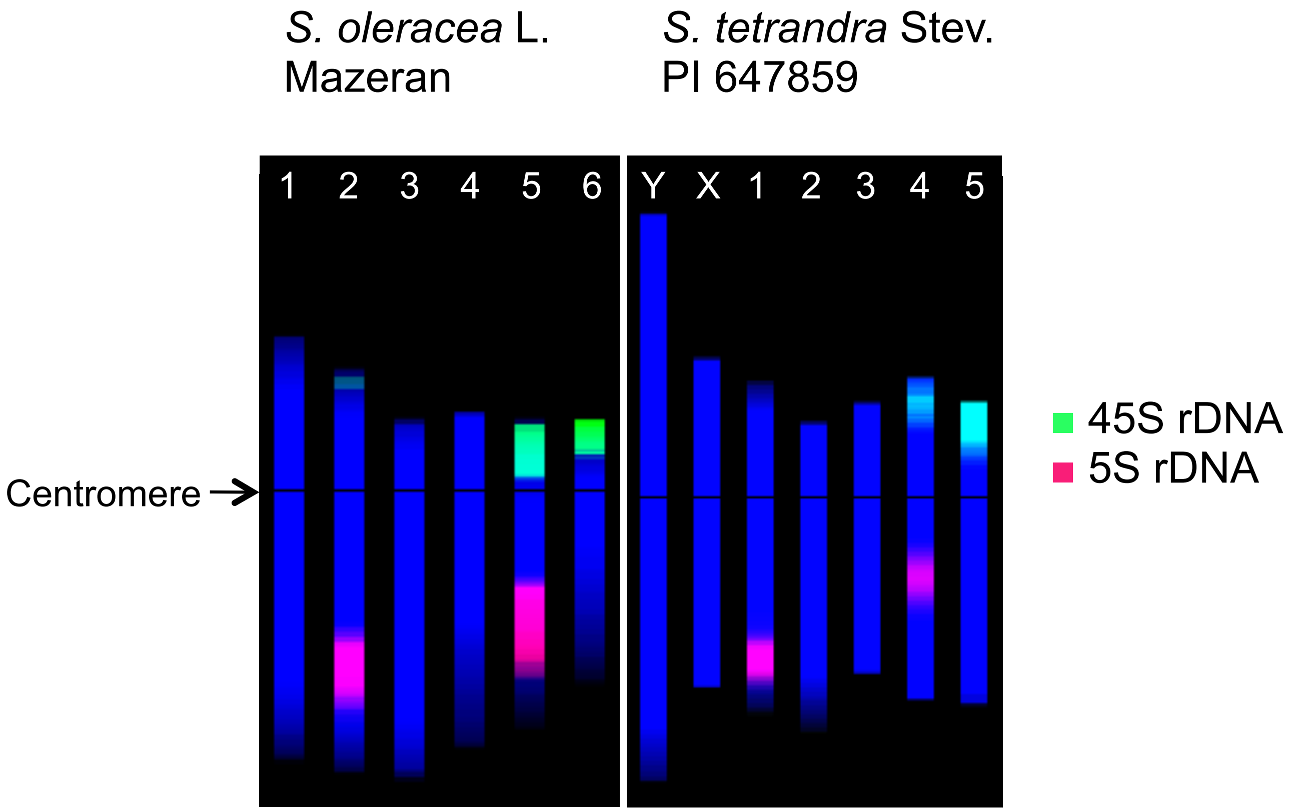

Figure S7. Color idiograms illustrating the metaphase chromosome complements of *S. oleracea* L. Mazeran and *S. tetrandra* Stev. PI 647859. The idiograms were constructed based on relative chromosome length, position of centromeres, and position and FISH signal intensity of 45S and 5S rDNA repeats. Autosomes of *S. tetrandra* Stev. PI 647859 were ordered according to their similarity in the possession of rDNA loci (45S and 5S) to those of *S. oleracea* L. Mazeran.

Table S1. Primer sequences used to amplify and sequence chloroplast intergenic spacers and internal transcribed spacer (ITS) regions of nuclear rRNA genes

Intergenic spacer	Primer sequence (5' to 3')
trnL-trnF	trnL-fw: AGGGGATATGGCGAAATC
	trnF-rv: GATTTGAACTGGTGACACGAG
rpl32-trnL	rpL32-F: GCAGTTCCAAAAAAGCGTACTTC[a]
	trnL: ATTTGTAAGATGCCATGCCG
trnV-ndhC	trnV: GTCTACGGTTCGAGTCCGTA[a]
	ndhC: TATTATTAGAAATGCCCAGAAAATATCATATTC[a]
ndhF-rpl32	ndhF: GAAAGGTATGATCCATGCATATT[a]
	rpL32-R: CCAATATCCCTTTTTTTTCCAA[a]
psbD-trnT	psbD: CTCCGTAACCAGTCATCCATA[a]
	trnT: CCCTTTTAACTCAGTGGTAG[b]
ITS	AC-ITS5: GGAAGGAGAAGTCGWAACARGG[c]
	ITS4: TCCTCCGCTTATTGATATGC[d]

[a]Modified from Shaw et al. (2007)

[b]Shaw et al. (2007)

[c]Fuentes-Bazan *et al.* (2012)

[d]White *et al.* (1990)

Table S2. Nucleotide sequences used for the phylogenetic analysis

Species	Accession, cultivar or line	Sequence ID No.					
		trnL-trnF	*rpl32-trnL*	*trnV-ndhC*	*ndhF-rpl32*	*psbD-trnT*	ITS
Spinacia oleracea L.	Ames 26244	AB935637*	AB935607*	LC009789*	LC009819*	LC009849*	AB935664*
	PI 173124	AB935638*	AB935608*	LC009790*	LC009820*	LC009850*	AB935665*
	PI 173972	AB935639*	AB935609*	LC009791*	LC009821*	LC009851*	AB935666*
	PI 181923	AB935640*	AB935610*	LC009792*	LC009822*	LC009852*	AB935667*
	PI 217425	AB935641*	AB935611*	LC009793*	LC009823*	LC009853*	AB935668*
	PI 604787	AB935642*	AB935612*	LC009794*	LC009824*	LC009854*	AB935669*
	PI 606707	AB935643*	AB935613*	LC009795*	LC009825*	LC009855*	AB935670*
	JP 25756	AB935644*	AB935614*	LC009796*	LC009826*	LC009856*	AB935671*
	JP 25763	AB935645*	AB935615*	LC009797*	LC009827*	LC009857*	AB935672*
	Mazeran	AB935646*	AB935616*	LC009798*	LC009828*	LC009858*	AB935673*
	Nippon	AB935647*	AB935617*	LC009799*	LC009829*	LC009859*	AB935674*
	SPI 588	AB935648*	AB935618*	LC009800*	LC009830*	LC009860*	AB935675*
	03-009	AB935649*	AB935619*	LC009801*	LC009831*	LC009861*	AB935676*
	86-36	AB935650*	AB935620*	LC009802*	LC009832*	LC009862*	AB935677*
	105-18	AB935651*	AB935621*	LC009803*	LC009833*	LC009863*	AB935678*
Spinacia turkestanica Ilj.	Ames 23666	HE577483	AB935622*	LC009804*	LC009834*	LC009864*	HE577346
	PI 494751	AB935652*	AB935623*	LC009805*	LC009835*	LC009865*	AB935679*
	PI 647863	AB935653*	AB935624*	LC009806*	LC009836*	LC009866*	AB935680*
	PI 604792	AB935654*	AB935625*	LC009807*	LC009837*	LC009867*	AB935681*
	PI 608713	AB935655*	AB935626*	LC009808*	LC009838*	LC009868*	AB935682*
	CGN 09594	AB935656*	AB935627*	LC009809*	LC009839*	LC009869*	AB935683*
	CGN 09597	AB935657*	AB935628*	LC009810*	LC009840*	LC009870*	AB935684*
Spinacia tetrandra Stev.	Ames 23664	HE577482	AB935629*	LC009811*	LC009841*	LC009871*	HE577345
	PI 608712	AB935658*	AB935630*	LC009812*	LC009842*	LC009872*	AB935685*
	PI 647859	AB935659*	AB935631*	LC009813*	LC009843*	LC009873*	AB935686*
	PI 647860	AB935660*	AB935632*	LC009814*	LC009844*	LC009874*	AB935687*
	PI 647861	AB935661*	AB935633*	LC009815*	LC009845*	LC009875*	AB935688*
Beta procumbens Chr. Sm.	sp541205-03	AB935663*	AB935636*	LC009816*	LC009846*	LC009876*	AB935691*
Beta vulgaris L.		HE577473	EF534108.1	EF534108.1	EF534108.1	EF534108.1	HE577334
Beta webbiana Moq.	Ames 4515	AB935662*	AB935634*	LC009818*	LC009848*	LC009878*	AB935689*

* Nucleotide sequences determined in the present study.

Table S3. Chromosome designation in spinach

Position of centromere	SAT	45S rDNA	5S rDNA	Sex[b]	Ellis and Janick (1960) [d]	Sugiyama and Suto (1964)[d], Ito *et al*. (2000)
Submedian				✔	1	1
Submedian	✔[a]	✔	✔		3	2
Subterminal					2	3
Subterminal					4	4
Subterminal	✔	✔	✔		5	5
Subterminal	✔[c]	✔			6	6

SAT, satellite chromosome; Sex, sex chromosome.

[a]A satellite on the short arm of the chromosome can be observed in a cultivar, though it is not seen in most spinach stocks (Iizuka and Janick, 1962).

[b]Ellis and Janick (1960).

[c]Sugiyama and Suto (1969) reported that both Chromosome 5 and 6 had satellites on their short arms.

[d]The chromosome designations proposed by Ellis and Janick (1960) and Sugiyama and Suto (1964) were consistent with each other except for the smallest submedian chromosome and the largest subterminal chromosome (Chromosome 2 and 3 in Sugiyama and Suto [1964], respectively). In this study, we follow the nomenclature proposed by Sugiyama and Suto (1964).

Table S4. Observed number of males and females from germplasm accessions of the wild *Spinacia* species

Species	Accession	Male	Female	Total
S. turkestanica Ilj.	Ames 23666	4	5	9
	PI 494751	10	10	20
	PI 647863	4	4	8
	PI 604792	6	4	10
	PI 608713	8	4	12
	CGN 09594	2	5	7
	CGN 09597	1	3	4
S. tetrandra Stev.	Ames 23664	8	8	16
	PI 608713	3	1	4
	PI 647859	11	7	18
	PI 647860	3	6	9
	PI 647861	6	4	10

Table S5. Pollen fertility of male plants in the parental spinach cultivar and *S. tetrandra* Stev. accessions of inter-group hybrids

Group	Species	Cultivar or accession	Pollen fertility
Group-1	*S. oleracea* L.	Nippon	95.8-98.9% (*N* = 8)
Group-2	*S. tetrandra* Stev.	PI 647859	91.4–98.1% (*N* = 5)
Group-2	*S. tetrandra* Stev.	PI 647860	95.3-99.1% (*N* = 3)
Group-2	*S. tetrandra* Stev.	PI 647861	93.4–99.2% (*N* = 4)

N, number of plants examined

Table S6. One-way ANOVA for the nuclear DNA amounts of 26 *Spinacia* plants presented in Figure 2

Source of variation	Sum of squares	d.f.	Mean square	*F*-statistics
Individuals	3.20212	25	0.12809	863.86853***
Error	0.01542	104	0.00015	
Total	3.21755	129		

***$P < 0.001$

Table S7. Post hoc Tukey's multiple comparisons of the nuclear DNA amounts between individuals.

Group	Species	Accession or		PI 173972	PI 181923		PI 217425		Nippon		Mazeran		SPI 588		PI 494751		PI 647863		Ames 23664		PI 608712		PI 647859		PI 647860		PI 647861	
		cultivar		♀	♂	♀	♂	♀	♂	♀	♂	♀	♂	♀	♂	♀	♂	♀	♂	♀	♂	♀	♂	♀	♂	♀	♂	♀
Group-1	*S. oleracea* L.	PI 173972	♂	NS	NS	NS	NS	NS	NS	NS	NS	NS	NS	NS	NS	NS	NS	NS	NS	NS	NS	NS	***	***	***	***	***	***
			♀		NS	NS	NS	NS	NS	NS	NS	NS	NS	NS	NS	NS	NS	NS	NS	NS	NS	NS	***	***	***	***	***	***
	S. oleracea L.	PI 181923	♂			NS	NS	NS	NS	NS	NS	NS	NS	NS	NS	NS	NS	NS	NS	NS	NS	NS	***	***	***	***	***	***
			♀				NS	NS	NS	NS	NS	NS	NS	NS	NS	NS	NS	NS	NS	NS	NS	NS	***	***	***	***	***	***
	S. oleracea L.	PI 217425	♂					NS	NS	NS	NS	NS	NS	NS	NS	NS	NS	NS	NS	NS	NS	NS	***	***	***	***	***	***
			♀						NS	NS	NS	NS	NS	NS	NS	NS	NS	NS	NS	NS	NS	NS	***	***	***	***	***	***
	S. oleracea L.	Nippon	♂							NS	NS	NS	NS	NS	NS	NS	NS	NS	NS	NS	NS	NS	***	***	***	***	***	***
			♀								NS	NS	NS	NS	NS	NS	NS	NS	NS	NS	NS	NS	***	***	***	***	***	***
	S. oleracea L.	Mazeran	♂									NS	NS	NS	NS	NS	NS	NS	NS	NS	NS	NS	***	***	***	***	***	***
			♀										NS	NS	NS	NS	NS	NS	NS	NS	NS	NS	***	***	***	***	***	***
	S. oleracea L.	SPI 588	♂											NS	NS	NS	NS	NS	NS	NS	NS	NS	***	***	***	***	***	***
			♀												NS	NS	NS	NS	NS	NS	NS	NS	***	***	***	***	***	***
	S. turkestanica Ilj.	PI 494751	♂													NS	NS	NS	NS	NS	NS	NS	***	***	***	***	***	***
			♀														NS	NS	NS	NS	NS	NS	***	***	***	***	***	***
	S. turkestanica Ilj.	PI 647863	♂															NS	NS	NS	NS	NS	***	***	***	***	***	***
			♀																NS	NS	NS	NS	***	***	***	***	***	***
	S. tetrandra Stev.	Ames 23664	♂																	NS	NS	NS	***	***	***	***	***	***
			♀																		NS	NS	***	***	***	***	***	***
	S. tetrandra Stev.	PI 608712	♂																			NS	***	***	***	***	***	***
			♀																				***	***	***	***	***	***
Group-2	*S. tetrandra* Stev.	PI 647859	♂																					***	NS	***	NS	***
			♀																						***	NS	***	NS
	S. tetrandra Stev.	PI 647860	♂																							***	NS	***
			♀																								***	NS
	S. tetrandra Stev.	PI 647861	♂																									***

NS, Not significant (≥ 0.05)

***Significant at the 0.001 level

Table S8. Single nucleotide polymorphism typing for the *ketohexokinase* (*khk*) locus in progeny plants from the cross between a male and a female plant in PI 647859, using dCAPS marker SP_0048

Progeny plants	SNP Genotypes		Total
	TT	TA	
Male	10	0	10
Female	0	23	23
Total	10	23	33
