# Supplementary material for: Evidence for a Common Origin of Homomorphic and Heteromorphic Sex Chromosomes in Distinct Spinacia Species
Source: G3 (Bethesda). 2015 Jun 5;5(8):1663–73. doi: 10.1534/g3.115.018671 (PMC4528323; doi:10.1534/g3.115.018671)
Supplement: Supporting Information [file supp_g3.115.018671_FigureS1.pdf]

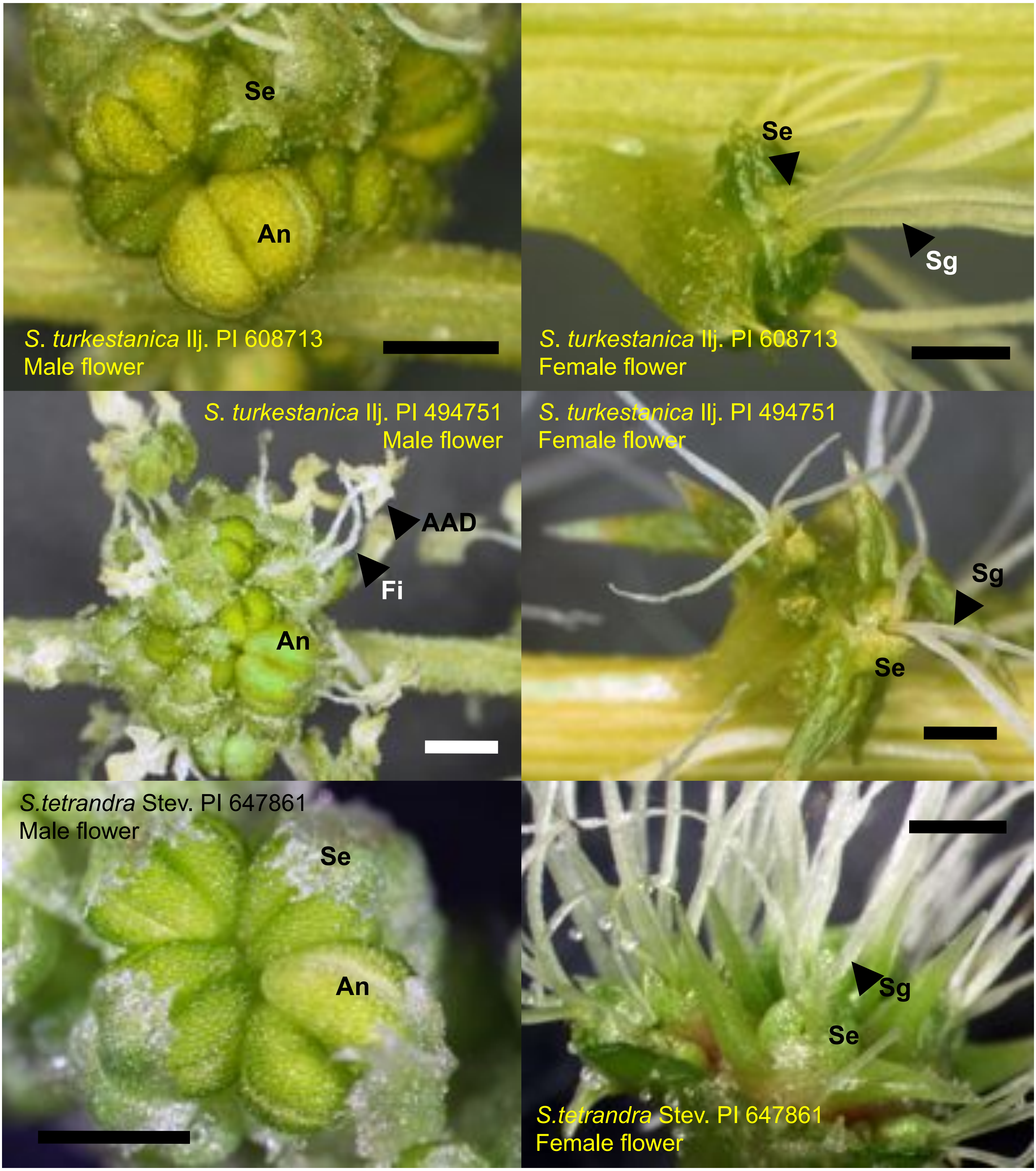

Figure S1. Floral morphology of *S. turkestanica* Ilj. and *S. tetrandra* Stev. Se, sepal; An, anther; AAD, anther after dehiscence; Fi, filament; Sg, stigma. Bar = 1.0 mm
