# Supplementary material for: Evidence for a Common Origin of Homomorphic and Heteromorphic Sex Chromosomes in Distinct Spinacia Species
Source: G3 (Bethesda). 2015 Jun 5;5(8):1663–73. doi: 10.1534/g3.115.018671 (PMC4528323; doi:10.1534/g3.115.018671)
Supplement: Supporting Information [file supp_g3.115.018671_FigureS2.pdf]

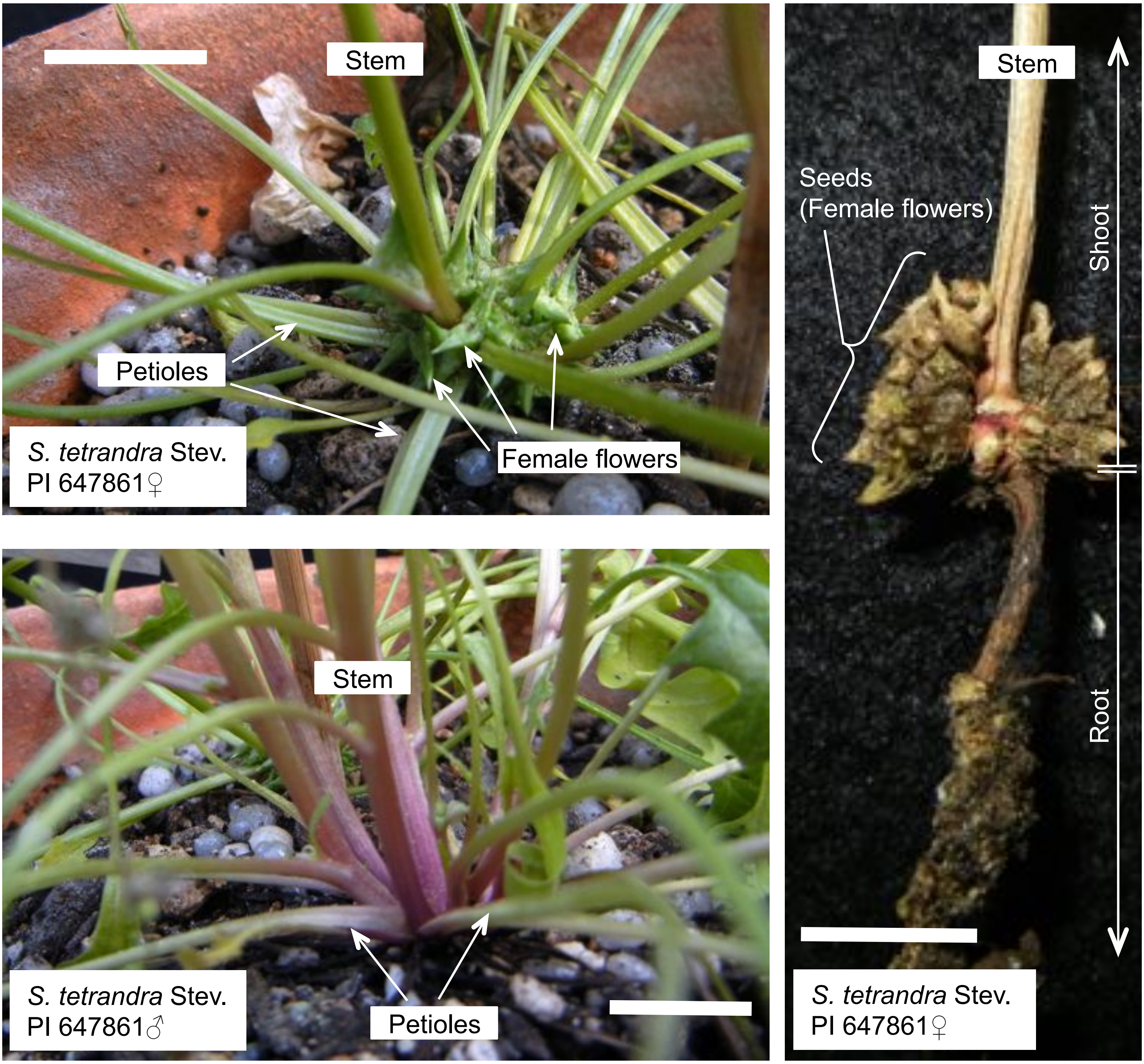

Figure S2. Axils of basal leaves of a male plant and female individuals from *S. tetrandra* Stev. PI 647861. Please note that flowers arose only from axils at base of a shoot of a female plant. Bar = 1.0 cm
