# Supplementary material for: Evidence for a Common Origin of Homomorphic and Heteromorphic Sex Chromosomes in Distinct Spinacia Species
Source: G3 (Bethesda). 2015 Jun 5;5(8):1663–73. doi: 10.1534/g3.115.018671 (PMC4528323; doi:10.1534/g3.115.018671)
Supplement: Supporting Information [file supp_g3.115.018671_FigureS3.pdf]

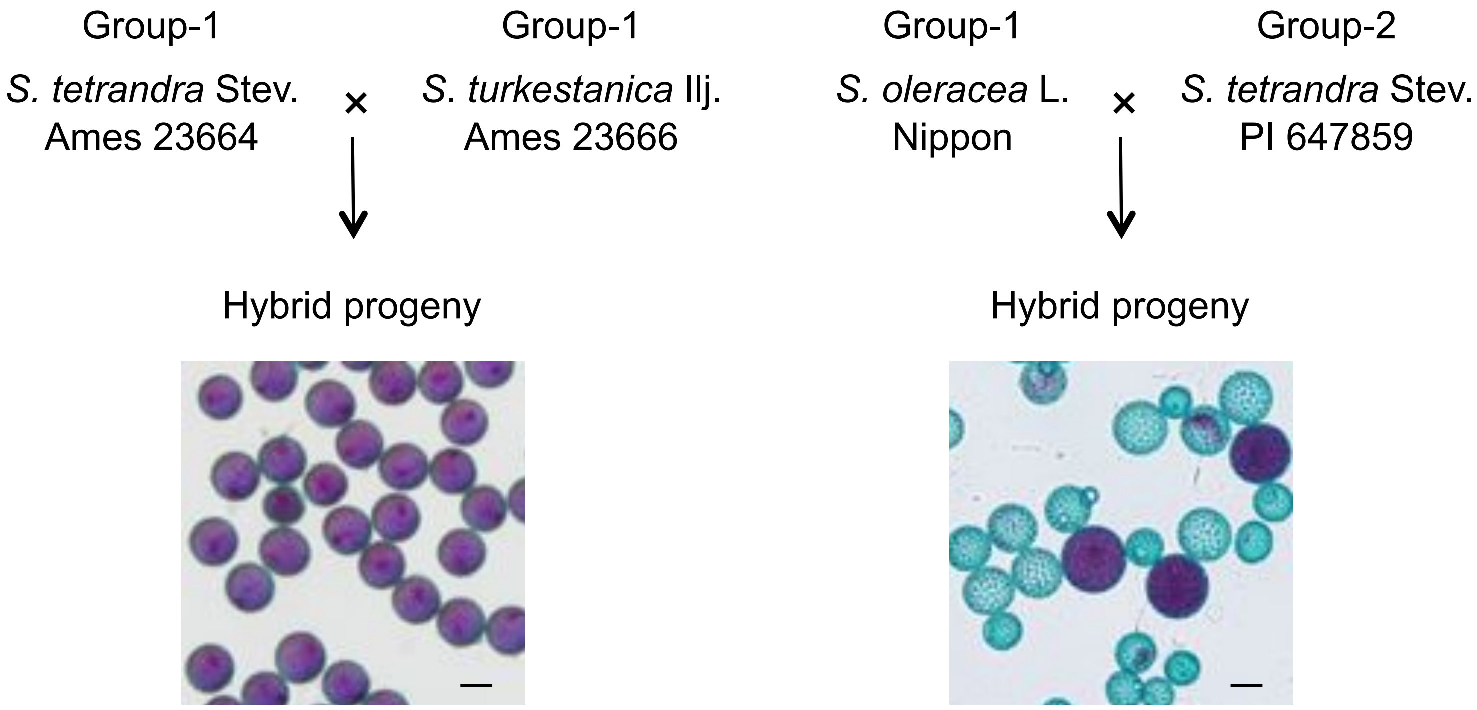

Figure S3. Pollen fertility of interspecific hybrids between *Spinacia* species. Pollen grains were stained with Alexander solution. Viable and non-viable pollen grains stained purple and pale-blue-green, respectively. Bars = 20 µm.
