# Supplementary material for: Evidence for a Common Origin of Homomorphic and Heteromorphic Sex Chromosomes in Distinct Spinacia Species
Source: G3 (Bethesda). 2015 Jun 5;5(8):1663–73. doi: 10.1534/g3.115.018671 (PMC4528323; doi:10.1534/g3.115.018671)
Supplement: Supporting Information [file supp_g3.115.018671_FigureS4.pdf]

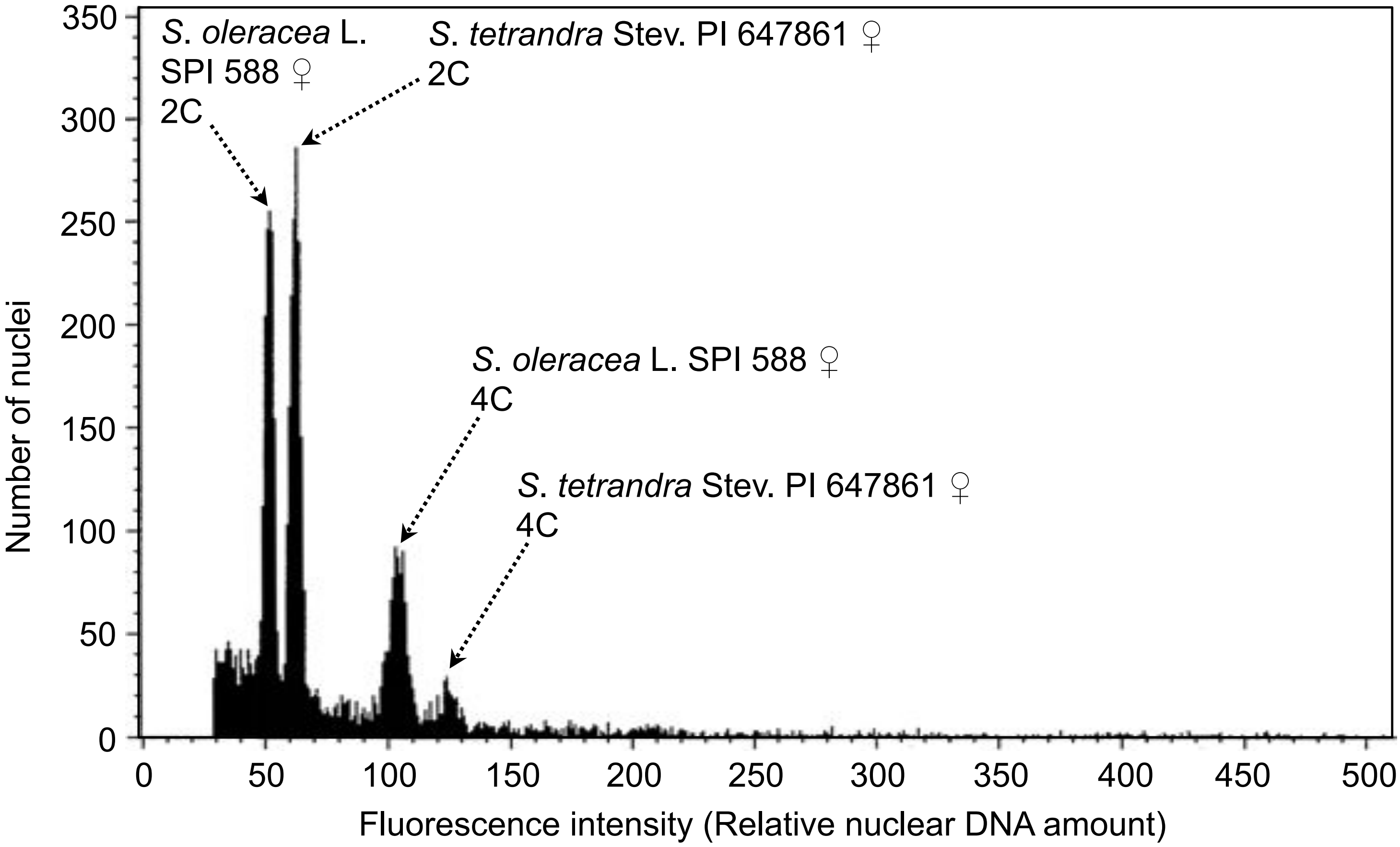

Figure S4. A histogram of relative DNA amount obtained after the flow cytometric analysis of nuclei isolated from female plants of *S. oleracea* L. SPI 588 and *S. tetrandra* Stev. PI 647861. Arrows indicate 2C peaks (nuclei of cells in the G0/G1 phase) and 4C peaks (G2/M phase).
