# Supplementary material for: Evidence for a Common Origin of Homomorphic and Heteromorphic Sex Chromosomes in Distinct Spinacia Species
Source: G3 (Bethesda). 2015 Jun 5;5(8):1663–73. doi: 10.1534/g3.115.018671 (PMC4528323; doi:10.1534/g3.115.018671)
Supplement: Supporting Information [file supp_g3.115.018671_FigureS6.pdf]

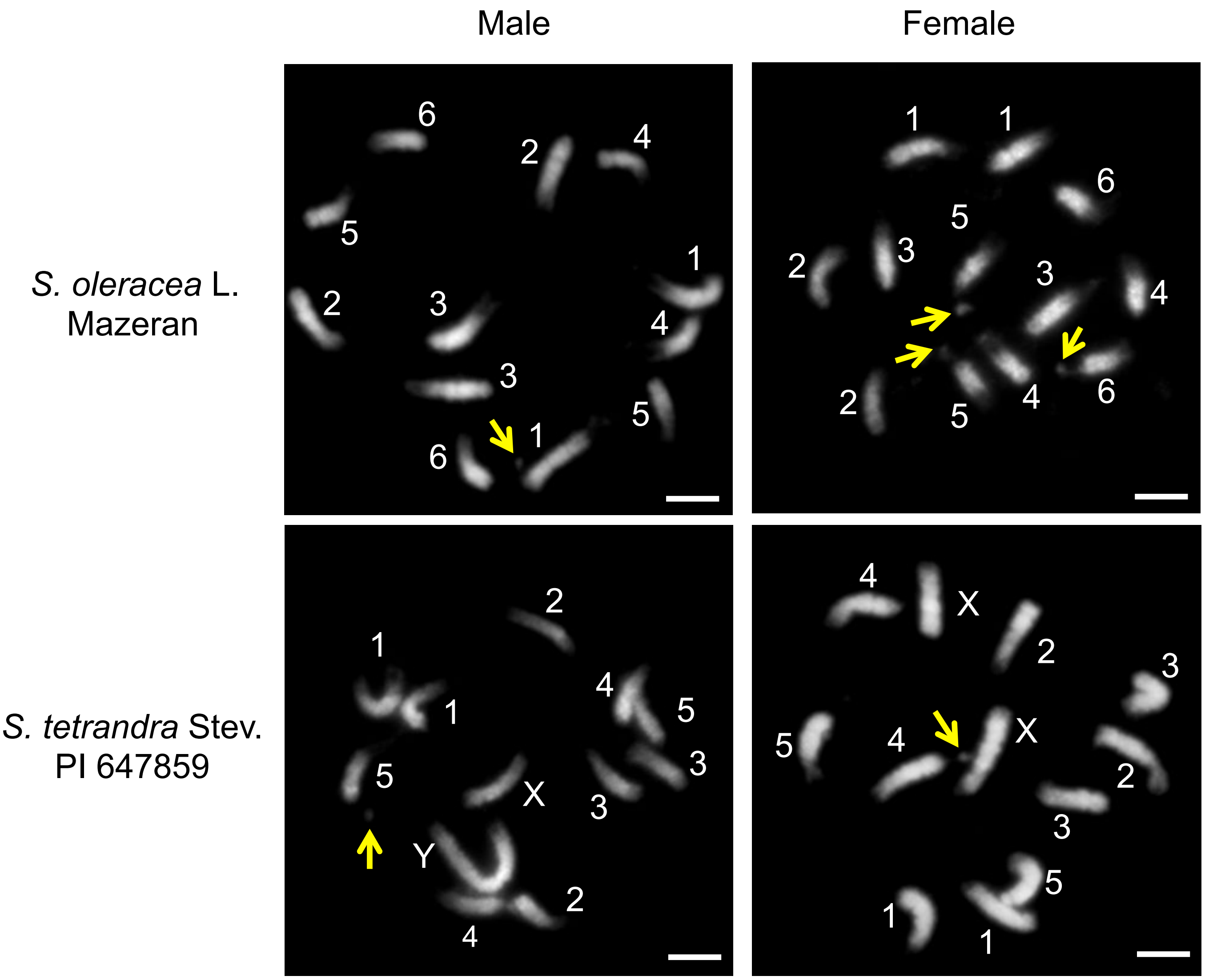

Figure S6. DAPI-stained mitotic prometaphase chromosomes in *S. oleracea* L. Mazeran and *S. tetrandra* Stev. PI 647859. Note that a heteromorphic chromosome pair (XY) can be found only in a male of *S. tetrandra* Stev. PI 647859. Arrows indicate satellites. Bars = 5µm.
