# Supplementary material for: Evidence for a Common Origin of Homomorphic and Heteromorphic Sex Chromosomes in Distinct Spinacia Species
Source: G3 (Bethesda). 2015 Jun 5;5(8):1663–73. doi: 10.1534/g3.115.018671 (PMC4528323; doi:10.1534/g3.115.018671)
Supplement: Supporting Information [file supp_g3.115.018671_FigureS7.pdf]

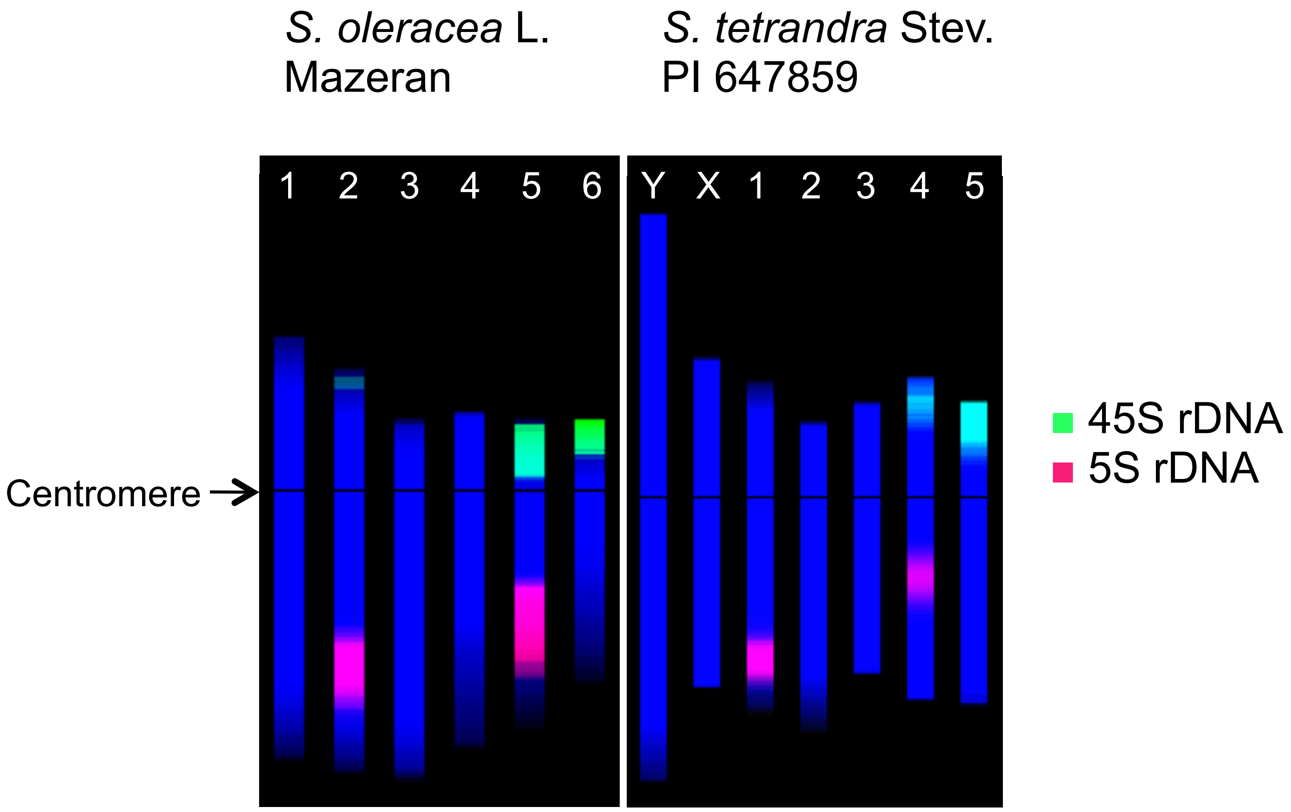

Figure S7. Color idiograms illustrating the metaphase chromosome complements of *S. oleracea* L. Mazeran and *S. tetrandra* Stev. PI 647859. The idiograms were constructed based on relative chromosome length, position of centromeres, and position and FISH signal intensity of 45S and 5S rDNA repeats. Autosomes of *S. tetrandra* Stev. PI 647859 were ordered according to their similarity in the possession of rDNA loci (45S and 5S) to those of *S. oleracea* L. Mazeran.
