# Supplementary material for: Evidence for a Common Origin of Homomorphic and Heteromorphic Sex Chromosomes in Distinct Spinacia Species
Source: G3 (Bethesda). 2015 Jun 5;5(8):1663–73. doi: 10.1534/g3.115.018671 (PMC4528323; doi:10.1534/g3.115.018671)
Supplement: Supporting Information [file supp_g3.115.018671_TableS1.pdf]

Table S1. Primer sequences used to amplify and sequence chloroplast intergenic spacers and internal transcribed spacer (ITS) regions of nuclear rRNA genes

Intergenic spacer	Primer sequence (5' to 3')
trnL-trnF	trnL-fw: AGGGGATATGGCGAAATC
	trnF-rv: GATTTGAACTGGTGACACGAG
rpl32-trnL	rpL32-F: GCAGTTCCAAAAAAGCGTACTTC[a]
	trnL: ATTTGTAAGATGCCATGCCG
trnV-ndhC	trnV: GTCTACGGTTCGAGTCCGTA[a]
	ndhC: TATTATTAGAAATGCCCAGAAAATATCATATTC[a]
ndhF-rpl32	ndhF: GAAAGGTATGATCCATGCATATT[a]
	rpL32-R: CCAATATCCCTTTTTTTTCCAA[a]
psbD-trnT	psbD: CTCCGTAACCAGTCATCCATA[a]
	trnT: CCCTTTTAACTCAGTGGTAG[b]
ITS	AC-ITS5: GGAAGGAGAAGTCGWAACARGG[c]
	ITS4: TCCTCCGCTTATTGATATGC[d]

[a]Modified from Shaw et al. (2007)

[b]Shaw et al. (2007)

[c]Fuentes-Bazan *et al.* (2012)

[d]White *et al.* (1990)
