# Supplementary material for: Evidence for a Common Origin of Homomorphic and Heteromorphic Sex Chromosomes in Distinct Spinacia Species
Source: G3 (Bethesda). 2015 Jun 5;5(8):1663–73. doi: 10.1534/g3.115.018671 (PMC4528323; doi:10.1534/g3.115.018671)
Supplement: Supporting Information [file supp_g3.115.018671_TableS2.pdf]

Table S2. Nucleotide sequences used for the phylogenetic analysis

Species	Accession, cultivar or line	Sequence ID No.					
		trnL-trnF	*rpl32-trnL*	*trnV-ndhC*	*ndhF-rpl32*	*psbD-trnT*	ITS
Spinacia oleracea L.	Ames 26244	AB935637*	AB935607*	LC009789*	LC009819*	LC009849*	AB935664*
	PI 173124	AB935638*	AB935608*	LC009790*	LC009820*	LC009850*	AB935665*
	PI 173972	AB935639*	AB935609*	LC009791*	LC009821*	LC009851*	AB935666*
	PI 181923	AB935640*	AB935610*	LC009792*	LC009822*	LC009852*	AB935667*
	PI 217425	AB935641*	AB935611*	LC009793*	LC009823*	LC009853*	AB935668*
	PI 604787	AB935642*	AB935612*	LC009794*	LC009824*	LC009854*	AB935669*
	PI 606707	AB935643*	AB935613*	LC009795*	LC009825*	LC009855*	AB935670*
	JP 25756	AB935644*	AB935614*	LC009796*	LC009826*	LC009856*	AB935671*
	JP 25763	AB935645*	AB935615*	LC009797*	LC009827*	LC009857*	AB935672*
	Mazeran	AB935646*	AB935616*	LC009798*	LC009828*	LC009858*	AB935673*
	Nippon	AB935647*	AB935617*	LC009799*	LC009829*	LC009859*	AB935674*
	SPI 588	AB935648*	AB935618*	LC009800*	LC009830*	LC009860*	AB935675*
	03-009	AB935649*	AB935619*	LC009801*	LC009831*	LC009861*	AB935676*
	86-36	AB935650*	AB935620*	LC009802*	LC009832*	LC009862*	AB935677*
	105-18	AB935651*	AB935621*	LC009803*	LC009833*	LC009863*	AB935678*
Spinacia turkestanica Ilj.	Ames 23666	HE577483	AB935622*	LC009804*	LC009834*	LC009864*	HE577346
	PI 494751	AB935652*	AB935623*	LC009805*	LC009835*	LC009865*	AB935679*
	PI 647863	AB935653*	AB935624*	LC009806*	LC009836*	LC009866*	AB935680*
	PI 604792	AB935654*	AB935625*	LC009807*	LC009837*	LC009867*	AB935681*
	PI 608713	AB935655*	AB935626*	LC009808*	LC009838*	LC009868*	AB935682*
	CGN 09594	AB935656*	AB935627*	LC009809*	LC009839*	LC009869*	AB935683*
	CGN 09597	AB935657*	AB935628*	LC009810*	LC009840*	LC009870*	AB935684*
Spinacia tetrandra Stev.	Ames 23664	HE577482	AB935629*	LC009811*	LC009841*	LC009871*	HE577345
	PI 608712	AB935658*	AB935630*	LC009812*	LC009842*	LC009872*	AB935685*
	PI 647859	AB935659*	AB935631*	LC009813*	LC009843*	LC009873*	AB935686*
	PI 647860	AB935660*	AB935632*	LC009814*	LC009844*	LC009874*	AB935687*
	PI 647861	AB935661*	AB935633*	LC009815*	LC009845*	LC009875*	AB935688*
Beta procumbens Chr. Sm.	sp541205-03	AB935663*	AB935636*	LC009816*	LC009846*	LC009876*	AB935691*
Beta vulgaris L.		HE577473	EF534108.1	EF534108.1	EF534108.1	EF534108.1	HE577334
Beta webbiana Moq.	Ames 4515	AB935662*	AB935634*	LC009818*	LC009848*	LC009878*	AB935689*

* Nucleotide sequences determined in the present study.
