# Supplementary material for: Evidence for a Common Origin of Homomorphic and Heteromorphic Sex Chromosomes in Distinct Spinacia Species
Source: G3 (Bethesda). 2015 Jun 5;5(8):1663–73. doi: 10.1534/g3.115.018671 (PMC4528323; doi:10.1534/g3.115.018671)
Supplement: Supporting Information [file supp_g3.115.018671_TableS3.pdf]

Table S3. Chromosome designation in spinach

Position of centromere	SAT	45S rDNA	5S rDNA	Sex[b]	Ellis and Janick (1960) [d]	Sugiyama and Suto (1964)[d], Ito *et al*. (2000)
Submedian				✔	1	1
Submedian	✔[a]	✔	✔		3	2
Subterminal					2	3
Subterminal					4	4
Subterminal	✔	✔	✔		5	5
Subterminal	✔[c]	✔			6	6

SAT, satellite chromosome; Sex, sex chromosome.

[a]A satellite on the short arm of the chromosome can be observed in a cultivar, though it is not seen in most spinach stocks (Iizuka and Janick, 1962).

[b]Ellis and Janick (1960).

[c]Sugiyama and Suto (1969) reported that both Chromosome 5 and 6 had satellites on their short arms.

[d]The chromosome designations proposed by Ellis and Janick (1960) and Sugiyama and Suto (1964) were consistent with each other except for the smallest submedian chromosome and the largest subterminal chromosome (Chromosome 2 and 3 in Sugiyama and Suto [1964], respectively). In this study, we follow the nomenclature proposed by Sugiyama and Suto (1964).
