# Supplementary material for: Evidence for a Common Origin of Homomorphic and Heteromorphic Sex Chromosomes in Distinct Spinacia Species
Source: G3 (Bethesda). 2015 Jun 5;5(8):1663–73. doi: 10.1534/g3.115.018671 (PMC4528323; doi:10.1534/g3.115.018671)
Supplement: Supporting Information [file supp_g3.115.018671_TableS4.pdf]

Table S4. Observed number of males and females from germplasm accessions of the wild *Spinacia* species

Species	Accession	Male	Female	Total
S. turkestanica Ilj.	Ames 23666	4	5	9
	PI 494751	10	10	20
	PI 647863	4	4	8
	PI 604792	6	4	10
	PI 608713	8	4	12
	CGN 09594	2	5	7
	CGN 09597	1	3	4
S. tetrandra Stev.	Ames 23664	8	8	16
	PI 608713	3	1	4
	PI 647859	11	7	18
	PI 647860	3	6	9
	PI 647861	6	4	10
