# Supplementary material for: Evidence for a Common Origin of Homomorphic and Heteromorphic Sex Chromosomes in Distinct Spinacia Species
Source: G3 (Bethesda). 2015 Jun 5;5(8):1663–73. doi: 10.1534/g3.115.018671 (PMC4528323; doi:10.1534/g3.115.018671)
Supplement: Supporting Information [file supp_g3.115.018671_TableS5.pdf]

Table S5. Pollen fertility of male plants in the parental spinach cultivar and *S. tetrandra* Stev. accessions of inter-group hybrids

Group	Species	Cultivar or accession	Pollen fertility
Group-1	*S. oleracea* L.	Nippon	95.8-98.9% (N = 8)
Group-2	*S. tetrandra* Stev.	PI 647859	91.4–98.1% (N = 5)
Group-2	*S. tetrandra* Stev.	PI 647860	95.3-99.1% (N = 3)
Group-2	*S. tetrandra* Stev.	PI 647861	93.4–99.2% (N = 4)

N, number of plants examined
