# Supplementary material for: Evidence for a Common Origin of Homomorphic and Heteromorphic Sex Chromosomes in Distinct Spinacia Species
Source: G3 (Bethesda). 2015 Jun 5;5(8):1663–73. doi: 10.1534/g3.115.018671 (PMC4528323; doi:10.1534/g3.115.018671)
Supplement: Supporting Information [file supp_g3.115.018671_TableS6.pdf]

Table S6. One-way ANOVA for the nuclear DNA amounts of 26 *Spinacia* plants presented in Figure 2

Source of variation	Sum of squares	d.f.	Mean square	*F*-statistics
Individuals	3.20212	25	0.12809	863.86853***
Error	0.01542	104	0.00015	
Total	3.21755	129		

***P < 0.001
