# Supplementary material for: Evidence for a Common Origin of Homomorphic and Heteromorphic Sex Chromosomes in Distinct Spinacia Species
Source: G3 (Bethesda). 2015 Jun 5;5(8):1663–73. doi: 10.1534/g3.115.018671 (PMC4528323; doi:10.1534/g3.115.018671)
Supplement: Supporting Information [file supp_g3.115.018671_TableS7.pdf]

Table S7. Post hoc Tukey's multiple comparisons of the nuclear DNA amounts between individuals.

Group	Species	Accession or		PI 173972	PI 181923		PI 217425		Nippon		Mazeran		SPI 588		PI 494751		PI 647863		Ames 23664		PI 608712		PI 647859		PI 647860		PI 647861	
		cultivar		♀	♂	♀	♂	♀	♂	♀	♂	♀	♂	♀	♂	♀	♂	♀	♂	♀	♂	♀	♂	♀	♂	♀	♂	♀
Group-1	*S. oleracea* L.	PI 173972	♂	NS	NS	NS	NS	NS	NS	NS	NS	NS	NS	NS	NS	NS	NS	NS	NS	NS	NS	NS	***	***	***	***	***	***
			♀		NS	NS	NS	NS	NS	NS	NS	NS	NS	NS	NS	NS	NS	NS	NS	NS	NS	NS	***	***	***	***	***	***
	S. oleracea L.	PI 181923	♂			NS	NS	NS	NS	NS	NS	NS	NS	NS	NS	NS	NS	NS	NS	NS	NS	NS	***	***	***	***	***	***
			♀				NS	NS	NS	NS	NS	NS	NS	NS	NS	NS	NS	NS	NS	NS	NS	NS	***	***	***	***	***	***
	S. oleracea L.	PI 217425	♂					NS	NS	NS	NS	NS	NS	NS	NS	NS	NS	NS	NS	NS	NS	NS	***	***	***	***	***	***
			♀						NS	NS	NS	NS	NS	NS	NS	NS	NS	NS	NS	NS	NS	NS	***	***	***	***	***	***
	S. oleracea L.	Nippon	♂							NS	NS	NS	NS	NS	NS	NS	NS	NS	NS	NS	NS	NS	***	***	***	***	***	***
			♀								NS	NS	NS	NS	NS	NS	NS	NS	NS	NS	NS	NS	***	***	***	***	***	***
	S. oleracea L.	Mazeran	♂									NS	NS	NS	NS	NS	NS	NS	NS	NS	NS	NS	***	***	***	***	***	***
			♀										NS	NS	NS	NS	NS	NS	NS	NS	NS	NS	***	***	***	***	***	***
	S. oleracea L.	SPI 588	♂											NS	NS	NS	NS	NS	NS	NS	NS	NS	***	***	***	***	***	***
			♀												NS	NS	NS	NS	NS	NS	NS	NS	***	***	***	***	***	***
	S. turkestanica Ilj.	PI 494751	♂													NS	NS	NS	NS	NS	NS	NS	***	***	***	***	***	***
			♀														NS	NS	NS	NS	NS	NS	***	***	***	***	***	***
	S. turkestanica Ilj.	PI 647863	♂															NS	NS	NS	NS	NS	***	***	***	***	***	***
			♀																NS	NS	NS	NS	***	***	***	***	***	***
	S. tetrandra Stev.	Ames 23664	♂																	NS	NS	NS	***	***	***	***	***	***
			♀																		NS	NS	***	***	***	***	***	***
	S. tetrandra Stev.	PI 608712	♂																			NS	***	***	***	***	***	***
			♀																				***	***	***	***	***	***
Group-2	*S. tetrandra* Stev.	PI 647859	♂																					***	NS	***	NS	***
			♀																						***	NS	***	NS
	S. tetrandra Stev.	PI 647860	♂																							***	NS	***
			♀																								***	NS
	S. tetrandra Stev.	PI 647861	♂																									***

NS, Not significant (≥ 0.05)

***Significant at the 0.001 level
