# Supplementary material for: Evidence for a Common Origin of Homomorphic and Heteromorphic Sex Chromosomes in Distinct Spinacia Species
Source: G3 (Bethesda). 2015 Jun 5;5(8):1663–73. doi: 10.1534/g3.115.018671 (PMC4528323; doi:10.1534/g3.115.018671)
Supplement: Supporting Information [file supp_g3.115.018671_TableS8.pdf]

Table S8. Single nucleotide polymorphism typing for the *ketohexokinase* (*khk*) locus in progeny plants from the cross between a male and a female plant in PI 647859, using dCAPS marker SP_0048

Progeny plants	SNP Genotypes		Total
	TT	TA	
Male	10	0	10
Female	0	23	23
Total	10	23	33
